# Identification and characterization of the pyridoxal 5’-phosphate allosteric site in *Escherichia coli* pyridoxine 5’-phosphate oxidase

**DOI:** 10.1016/j.jbc.2021.100795

**Published:** 2021-05-18

**Authors:** Anna Barile, Theo Battista, Annarita Fiorillo, Martino Luigi di Salvo, Francesco Malatesta, Angela Tramonti, Andrea Ilari, Roberto Contestabile

**Affiliations:** 1 Istituto di Biologia e Patologia Molecolari, Consiglio Nazionale delle Ricerche, Roma, Italy; 2 Dipartimento di Scienze Biochimiche “A. Rossi Fanelli”, Istituto Pasteur Italia - Fondazione Cenci Bolognetti, Sapienza Università di Roma, Roma, Italy

**Keywords:** pyridoxine 5’-phosphate oxidase, vitamin B_6_ metabolism, bacterial metabolism, enzyme inhibitor, pyridoxal phosphate, allosteric regulation, crystal structure, asymmetry, DSF, differential scanning fluorimetry, *e*PNPO, *Escherichia coli* PNPO, PLP, pyridoxal 5’-phosphate, PNP, pyridoxine 5’-phosphate, PNPO, pyridoxine 5’-phosphate oxidase

## Abstract

Pyridoxal 5’-phosphate (PLP), the catalytically active form of vitamin B_6_, plays a pivotal role in metabolism as an enzyme cofactor. PLP is a very reactive molecule and can be very toxic unless its intracellular concentration is finely regulated. In *Escherichia coli*, PLP formation is catalyzed by pyridoxine 5’-phosphate oxidase (PNPO), a homodimeric FMN-dependent enzyme that is responsible for the last step of PLP biosynthesis and is also involved in the PLP salvage pathway. We have recently observed that *E. coli* PNPO undergoes an allosteric feedback inhibition by PLP, caused by a strong allosteric coupling between PLP binding at the allosteric site and substrate binding at the active site. Here we report the crystallographic identification of the PLP allosteric site, located at the interface between the enzyme subunits and mainly circumscribed by three arginine residues (Arg23, Arg24, and Arg215) that form an “arginine cage” and efficiently trap PLP. The crystal structure of the PNPO–PLP complex, characterized by a marked structural asymmetry, presents only one PLP molecule bound at the allosteric site of one monomer and sheds light on the allosteric inhibition mechanism that makes the enzyme-substrate–PLP ternary complex catalytically incompetent. Site-directed mutagenesis studies focused on the arginine cage validate the identity of the allosteric site and provide an effective means to modulate the allosteric properties of the enzyme, from the loosening of the allosteric coupling (in the R23L/R24L and R23L/R215L variants) to the complete loss of allosteric properties (in the R23L/R24L/R21L variant).

Pyridoxal 5’-phosphate (PLP) acts as cofactor for over 150 enzymes (1, 2) involved in a number of metabolic pathways such as the synthesis, transformation, and degradation of amines and amino acids; supply of one carbon units; transsulfuration; synthesis of tetrapyrrolic compounds (including heme) and polyamines; and biosynthesis and degradation of neurotransmitters. In the cell, PLP is supplied to apoenzymes through either biosynthesis or recycling of B_6_ vitamers coming from the environment and protein turnover. The latter route is the only one available in organisms that cannot synthesize PLP, such as humans, and they obtain it through a salvage pathway (Fig. 1) catalyzed by the enzymes pyridoxal kinase, pyridoxine 5’-phosphate oxidase (PNPO; EC 1.4.3.5), and either specific or nonspecific phosphatases (3). By using FMN as cofactor, PNPO catalyzes the oxidation of both pyridoxine 5’-phosphate (PNP) and pyridoxamine 5’-phosphate to PLP, reducing molecular oxygen to hydrogen peroxide. With the *Escherichia coli* PNPO (*e*PNPO), the specificity constant for PNP is 50-fold higher than that for pyridoxamine 5’-phosphate (4). In *E. coli*, which synthesizes PLP through the so-called DXP-dependent pathway, *e*PNPO (encoded by the *pdxH* gene) plays the dual role of salvage pathway enzyme and of last enzyme in PLP biosynthesis (Fig. 1). This is the reason why the reaction catalyzed by *e*PNPO is a key point in PLP homeostasis.

**Figure 1:**
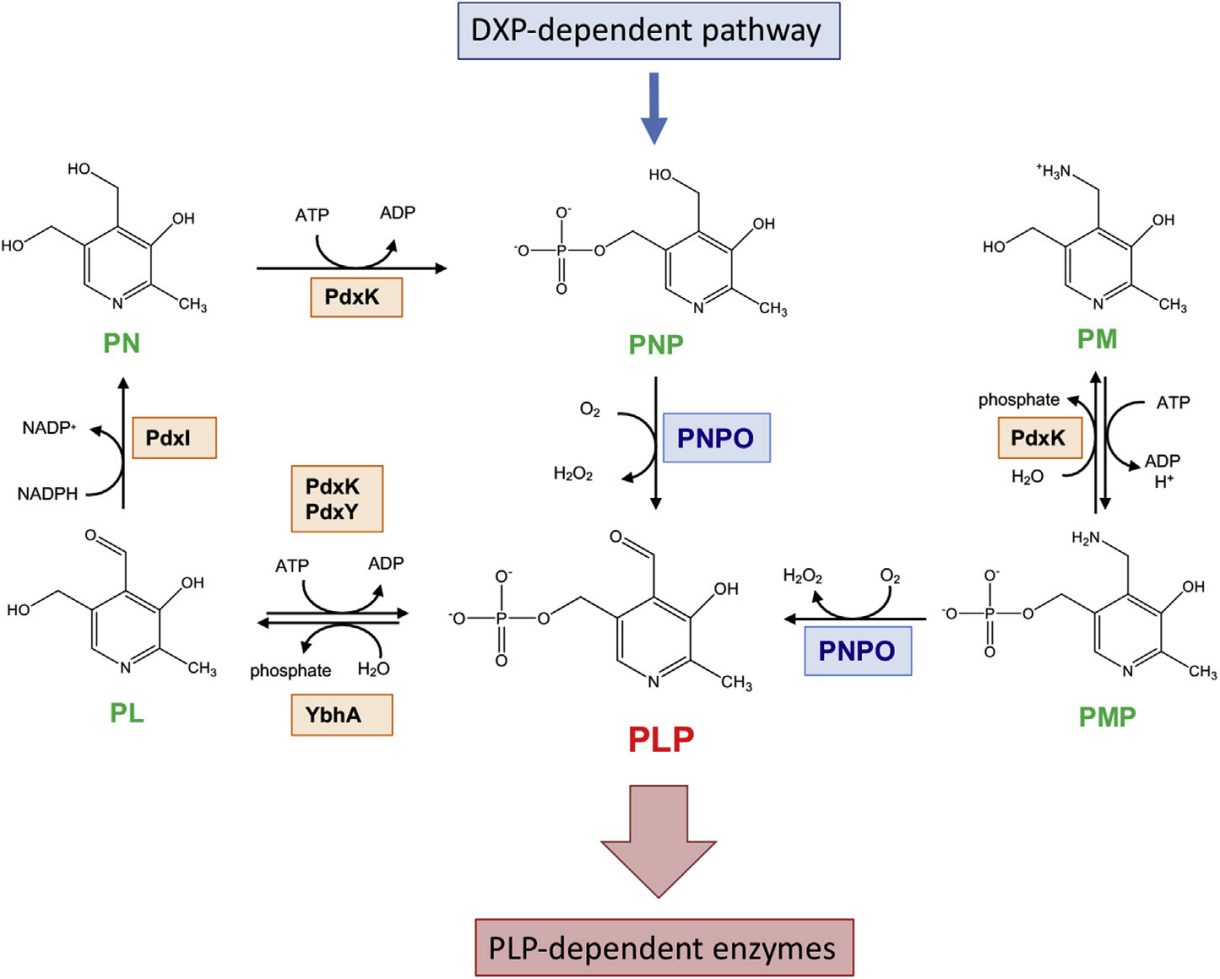
**General scheme of vitamin B**_**6**_**metabolism in *Escherichia coli*.** In *E. coli*, pyridoxine 5’-phosphate (PNP) biosynthesis takes place through the so-called deoxyxylulose 5-phosphate (DXP)-dependent pathway (18). Pyridoxal 5’-phosphate (PLP) is obtained by the oxidation of PNP catalyzed by pyridoxine 5’-phosphate oxidase (PNPO). A PLP salvage pathway is also present that involves pyridoxal kinase 1 (PdxK), which catalyzes the phosphorylation of pyridoxal (PL), pyridoxine (PN) and pyridoxamine (PM), pyridoxal kinase 2 (PdxY), which specifically phosphorylates PL (19), and a specific PLP phosphatase (YbhA). Pyridoxal reductase (PdxI) catalyzes the NADPH-dependent reduction of PL into PN (20). In plants, fungi, and most bacteria, PLP is directly synthesized through the DXP-independent pathway (18).

We have recently demonstrated (5) that *e*PNPO undergoes an allosteric feedback inhibition by PLP, through a mechanism in which binding of substrate at the active site and binding of PLP at an allosteric site negatively affect each other to a large extent (Fig. 2*A*). Such a strong allosteric coupling is also reflected in the lack of catalytic competence of the enzyme–PNP–PLP ternary complex (PES in Fig. 2*A*) and in the lack of capability by the enzyme–PLP complex (PE, in which PLP is bound at the allosteric site) to bind a second PLP molecule at the active site (at least in the range of PLP concentration used in kinetics measurements). Since binding of PLP at the allosteric site takes place with higher affinity than binding at the active site, PLP binding at the active site of the free enzyme does not occur.

**Figure 2:**
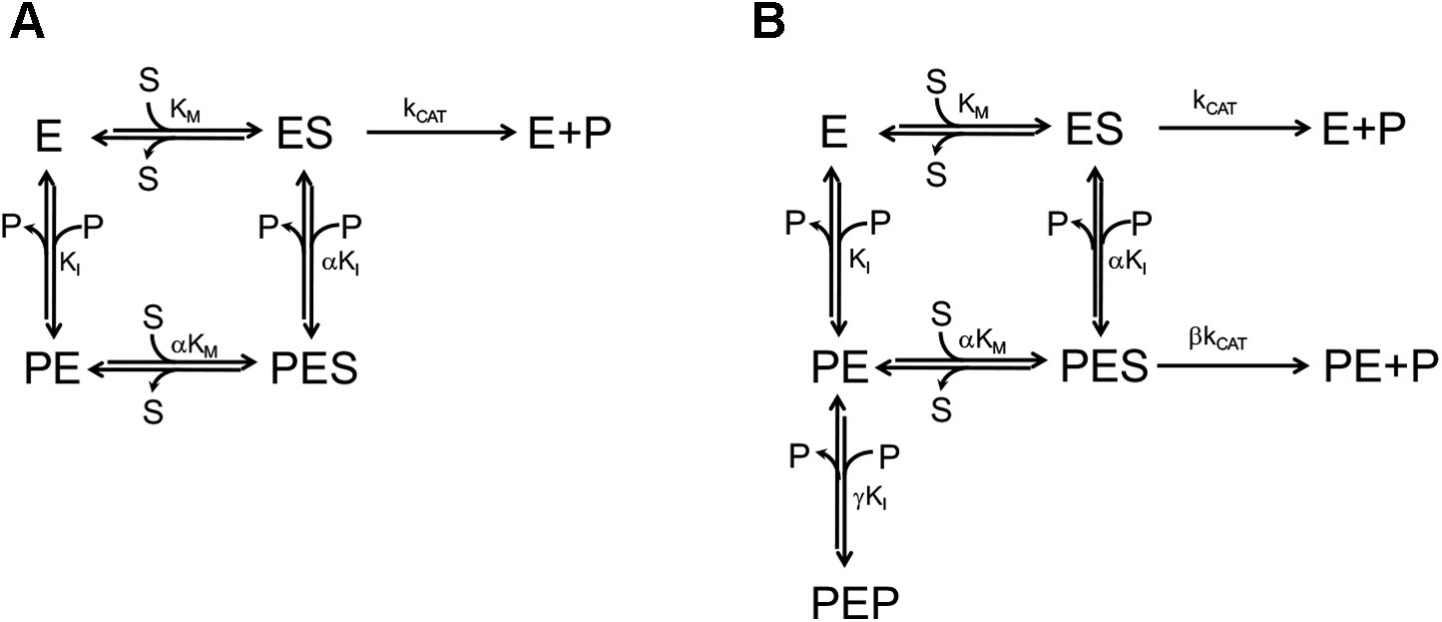
**Rapid equilibrium kinetics models.***A*, rapid equilibrium kinetics model of the linear mixed-type inhibition observed in *Escherichia coli* pyridoxine 5’-phosphate oxidase (5), in which the free enzyme (E) is able to bind both the pyridoxine 5’-phosphate substrate (S) and the pyridoxal 5’-phosphate (PLP) product (P) at the same time, respectively, at the active site and at an allosteric site. Binding of substrate and PLP affect each other by increasing K_I_ and *K*_M_ of an α coefficient, whose value is about 12. The PES ternary complex is catalytically inactive. *B*, rapid equilibrium kinetics model corresponding to the parabolic inhibition observed with human pyridoxine 5’-phosphate oxidase (6). This model is similar to that shown in *A*; however, in this case, the allosteric coupling between the allosteric site and the active site is weaker (the α value is about 3) and the PES ternary complex is catalytically active, although *k*_CAT_ is reduced by a β coefficient (equal to 0.15). Moreover, the PE complex formed upon binding of PLP at the allosteric site is able to bind a second PLP molecule at the active site (forming the dead-end PEP complex) with a K_I_ that is increased of a γ coefficient (equal to 6.6).

PLP allosteric inhibition is also observed with human PNPO (6). However, in this case the coupling between the allosteric site and the active site is weaker, so that the PES complex maintains a partial catalytic activity and PLP can also bind at the active site, forming an enzyme complex with two PLP molecules (PEP in Fig. 2*B*).

The PLP allosteric site of *e*PNPO most likely corresponds to the secondary “PLP tight binding site” previously revealed by other authors (7) when they observed that, if the enzyme is incubated with PLP and then passed through a size exclusion chromatography (SEC) column, it retains PLP. This observation is due to the fact that PLP binds tightly at a secondary site, distinct from the active site, as demonstrated by a quadruple *e*PNPO variant whose active site is impaired and that preserves this property (5). In the cell, PLP tightly bound to this secondary site of *e*PNPO is protected from the environment where, owing to the high reactivity of its aldehyde group, it would readily combine with all sorts of amines and thiols present in the cell; at the same time, it can be transferred to PLP-dependent apoenzymes, suggesting that *e*PNPO may play a role in the intracellular PLP delivery process (7).

A crystal structure of *e*PNPO (Protein Data Bank [PDB] code: 1G79), obtained from crystals soaked in a solution containing a large concentration of PLP (40 mM), revealed a putative secondary PLP-binding site located at the protein surface, about 11 Å from the active site (8). However, so far the real correspondence of this binding site to the PLP tight binding site has not been experimentally confirmed. In this crystal structure, in which PLP is also bound at the active site, PLP at the secondary site is present in two positions, with an occupancy ratio of 0.7 and 0.3, respectively. In the higher occupancy position, the PLP ring is sandwiched between the side chains of Phe177 and Lys145, the pyridine nitrogen interacts with Asn84, and the phosphate group interacts with the amino group of Lys145 (8) (Fig. S1). PLP in the lower occupancy position has a much weaker interaction with the protein and might result from crystal packing (8). Here we demonstrate, through site-directed mutagenesis experiments, that this putative site is not involved in the tight, allosteric binding of PLP. On the other hand, we employed a quadruple *e*PNPO variant (K72I/Y129F/R133L/H199A), with an impaired active site, to obtain a crystal structure of the enzyme in which PLP is bound at a novel secondary site. We show that this coincides with the allosteric PLP tight binding site.

## Results

### The secondary site indicated by previous crystallographic investigations does not correspond to the allosteric PLP tight binding site

The actual involvement of the putative secondary site identified by previous crystallographic studies (8) in the allosteric binding of PLP was tested through site-directed mutagenesis. A double variant (K145A/F177A) and two triple variants (N84A/K145A/F177A and N84W/K145A/F177A) of *e*PNPO (Fig. S1) were produced and characterized. Initially, these variant forms were analyzed with respect to their structure, thermal stability, and catalytic properties, to confirm that the introduced replacements had not significantly altered the native structure of the enzyme. Far-UV CD spectra (Fig. S2) indicated that no substantial alterations in the secondary structure were caused by the variations. Differential scanning fluorimetry (DSF) measurements were carried out to determine and compare melting temperature (*T*_m_) values of variant and wildtype enzymes. The obtained denaturation curves (Fig. S3*A*) were fitted to a sigmoidal equation (Equation 1) to obtain *T*_m_ values. The wildtype enzyme shows a *T*_m_ of 70.0 °C ± 0.04 deg. C. The double variant form shows a similar *T*_m_ value, whereas the melting temperature of the triple variants is about 2 °C lower (Table 1). These results indicate that the variations did not drastically alter the overall structure of the enzyme.

**Table 1:** Parameters obtained from the characterization of ePNPO variants

Enzyme form	*T*_m_ (°C)	a*K*_M_ (μM)	a*k*_CAT_ (s^−1^)	bK_D_^PLP^ (μM)	cPLP (%)
Wildtype	70.0 ± 0.04	1.6 ± 0.3	0.25 ± 0.03	0.15 (5)	69 ± 19
K145A/F177A	70.9 ± 0.05	2.4 ± 0.5	0.17 ± 0.01	0.47 ± 0.06	72 ± 8
N84A/K145A/F177A	66.7 ± 0.05	3.2 ± 0.1	0.07 ± 0.02	0.36 ± 0.05	73 ± 10
N84W/K145A/F177A	66.6 ± 0.06	3.1 ± 0.2	0.05 ± 0.01	0.33 ± 0.07	73 ± 19
R23L	70.0 ± 0.1	1.7 ± 0.7	0.09 ± 0.02	0.58 ± 0.12	38 ± 11
R215L	69.0 ± 0.09	2.6 ± 0.8	0.05 ± 0.02	1.28 ± 0.22	22 ± 7
R23L-R24L	71.1 ± 0.04	2.1 ± 0.4	0.19 ± 0.03	1.00 ± 0.09	30 ± 8
R23L/R215L	68.9 ± 0.03	3.2 ± 0.7	0.05 ± 0.02	1.18 ± 0.17	18 ± 9
R23L-R24L-R215L	68.0 ± 0.05	3.4 ± 0.2	0.09 ± 0.01	1.03 ± 0.18	19 ± 3

a Determined in Tris buffer from kinetic analysis.

b Determined from fluorimetric PLP binding equilibrium measurements.

c Determined from PLP retention measurements.

The catalytic properties of the *e*PNPO variants were analyzed in 50 mM Tris-HCl buffer, pH 7.6, at 37 °C, using PNP as substrate. In these conditions, the produced PLP readily forms an aldimine complex with Tris, whose absorbance at 414 nm is followed to determine the initial velocity of the PNPO reaction (9). Therefore, in Tris buffer PLP does not accumulate in the solvent and does not inhibit the enzyme activity by binding at the allosteric site (5). Initial velocity data as a function of substrate concentration were analyzed using the Michaelis–Menten equation, determining *k*_CAT_ and *K*_M_ values compared in Table 1. Although *K*_M_ values obtained with the variant enzymes are similar to that of wildtype *e*PNPO, *k*_CAT_ values are somewhat lower; in particular, in the case of the triple N84W/K145A/F177A variant, *k*_CAT_ is about five times lower than that of wildtype *e*PNPO. Nevertheless, it is evident that the variations did not perturb the functional properties of the enzyme.

Binding of PLP to *e*PNPO variants was also analyzed using the spectrofluorometric method previously employed for the wildtype enzyme (5), which is based on the resulting increase of FMN fluorescence emission. As we have previously demonstrated (5), PLP does not bind at the active site of wildtype *e*PNPO, most probably because binding at the allosteric site, which takes place with higher affinity, causes a conformational change that prevents binding at the active site. Therefore, the fluorimetric method only reveals binding of PLP at the allosteric site. PLP binding curves obtained with *e*PNPO variants (Fig. S5*A*) were fit to the quadratic Equation 2 (see Experimental procedures section), obtaining dissociation constant (K_D_) values (Table 1) that are moderately higher (0.33–0.47 μM) than that previously reported for wildtype *e*PNPO (0.15 μM) (5). This result is inconsistent with a role for the mutated residues in PLP binding at the allosteric site.

It is known by previous experiments on wildtype *e*PNPO that, when the protein is incubated with PLP and then passed through an SEC column, it retains PLP tightly bound to it (7). This happens even when the active site is unable to bind PLP as a consequence of multiple variations (5), demonstrating that PLP binds tightly at an allosteric site. All *e*PNPO variants (100 μM) were incubated with an equimolar amount of PLP. Then, they were passed through an SEC column and the stoichiometry of protein-bound PLP with respect to protein subunits was determined. The percentage of protein-bound PLP was 72 ± 8, 73 ± 10, and 73 ± 19, with the K145A/F177A, N84A/K145A/F177A and N84W/K145A/F177A *e*PNPO variants, respectively (Table 1). Such values are similar to that obtained with the wildtype enzyme (69 ± 19%), strongly suggesting that the putative secondary PLP-binding site indicated by previous crystallographic studies does not correspond to the allosteric tight binding site.

### Identification of the allosteric PLP-binding site of *E. coli* PNPO through crystallographic studies

As explained in the introduction, a crystal structure of *e*PNPO in which PLP is bound at both the active site and a secondary site has been previously reported by other authors (8). This structure was obtained by soaking the native crystals in a highly concentrated solution of PLP (40 mM). Under these conditions, it would be expected that PLP binds to the high-affinity allosteric site, the secondary site found by Safo and collaborators (8) and the active site; therefore, it is surprising that the high-affinity allosteric site had not been revealed in previous crystallographic studies, unless the allosteric site was occluded by crystal packing.

We carried out several cocrystallization trials with wildtype *e*PNPO and PLP in different conditions; however, we either did not obtain crystals or obtained crystals in which PLP was bound at the active site only. Therefore, we decided to change strategy and use a variant form of *e*PNPO in which the active site is impaired and not able to bind PLP. We recently showed that an *e*PNPO quadruple variant (K72I/Y129F/R133L/H199A; *e*PNPOqm), which has a drastically reduced capability to bind PNP (and therefore PLP) at the active site, is still able to bind PLP at the allosteric site (5). We crystallized this quadruple variant in both the absence of PLP (*e*PNPOqm) in P3_1_ 2 1 space group and presence of PLP (PLP-*e*PNPOqm) in P6_1_ space group. The three-dimensional structures of both crystal forms were solved (Table 2).

The final model of *e*PNPOqm was refined to 1.56 Å resolution; the asymmetric unit contains a 212-residue subunit (residues 7–218; the entire polypeptide chain is made by 218 residues), one FMN molecule, two sulphate ions, one phosphate ion, and 174 water molecules. The second subunit of the dimer was generated by the crystallographic 2-fold axis.

The final model of the PLP-bound *e*PNPOqm (PLP-*e*PNPOqm) was refined to 2.42 Å resolution; the asymmetric unit contains a 353-residue dimer (residues/chain: 17/A–126/A; 176/A–218/A; 14/B–166/B; 170/B–218/B), one FMN molecule per subunit, one sulphate ion in subunit A, one PLP molecule with 0.81 occupancy in subunit B, and 45 water molecules.

The quadruple mutant conserves the structural elements of the wildtype protein: five alpha helices, numbered from H1 to H5, and ten β strands. In addition, in the PLP-free *e*PNPOqm the N-terminal region is also visible, folded in an additional α helix (H0, residues 5–18).

**Figure 3:**
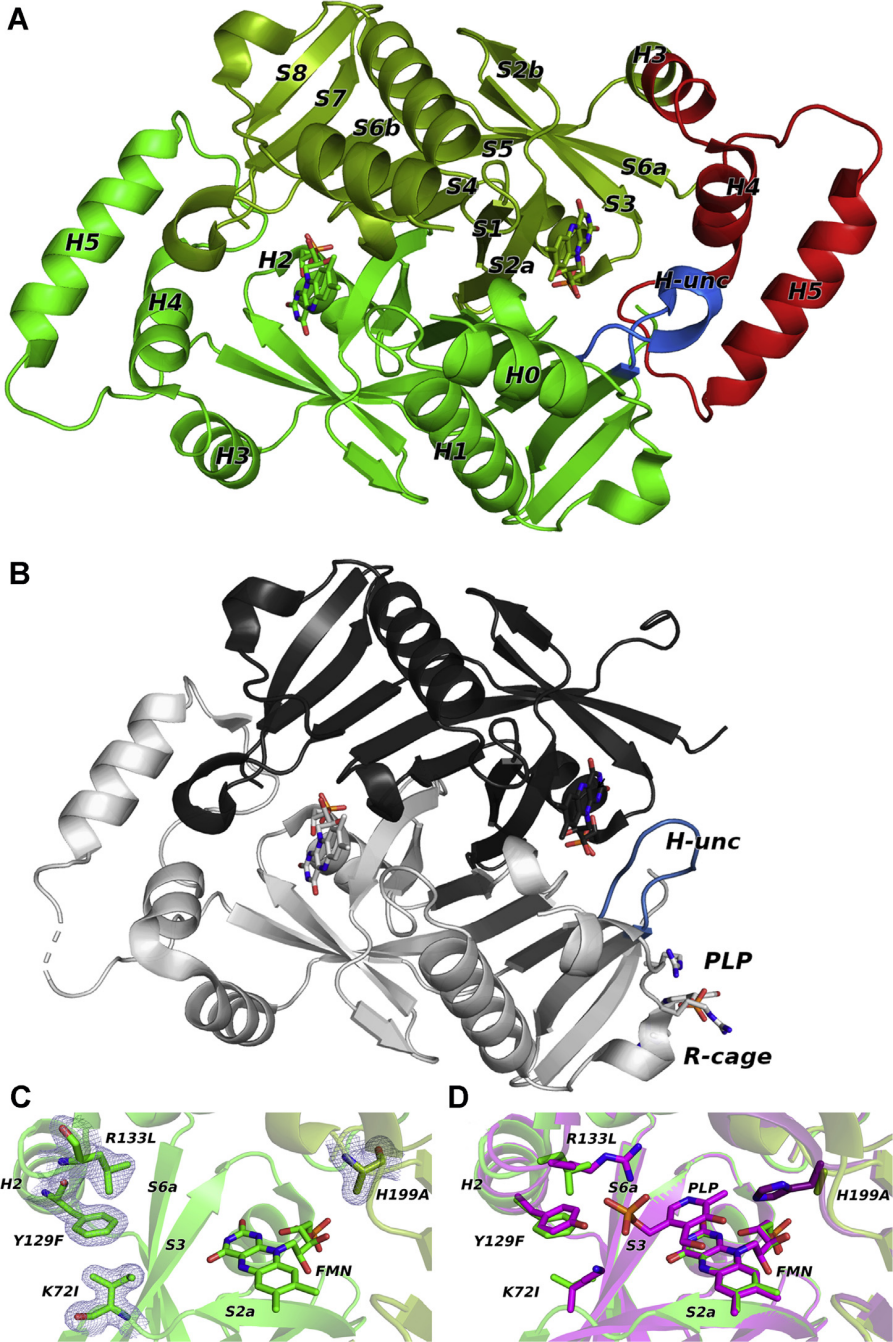
**Structures of *e*PNPOqm in the free and pyridoxal 5’-phosphate (PLP)-bound forms.***A*, overall fold of the *e*PNPOqm without PLP crystallized in the P3_1_ 2 1 space group. The second subunit of the dimer is generated by the crystallographic 2-fold axis. The two symmetric subunits of the dimer are colored in *light* (subunit A) and *dark* (subunit B) *green*. The secondary structure elements are labeled, following the numbering of Safo *et al.* (10). The structural elements that undergo the biggest structural changes upon PLP binding are colored in *red* (H3, H4, H5) and in *blue* (H-unc). *B*, overall fold of *e*PNPOqm in the PLP-bound form, crystallized in the P6_1_ space group. The two asymmetric subunits of the dimer are colored in *dark* (subunit A) and *light* (subunit B) *gray*; the H-unc structural element is colored in *blue*. PLP bound to subunit B and the arginine residues of the Arg cage are represented as *sticks*. *C*, blow-up of one of the mutated active sites of the *e*PNPOqm: K72I, Y129F, R133L, H199A 2Fo-Fc maps, contoured at 1σ, is displayed in *blue*. *D*, a structural alignment of *e*PNPOqm with a wildtype *E. coli* pyridoxine 5’-phosphate oxidase structure in which PLP is bound at the active site (Protein Data Bank code: 1G76, in *purple*) shows how the four variations can effectively prevent PLP binding at the active site by removing important polar and hydrophobic contacts.

The subunit structure can be described, as reported by Safo *et al.*, (10) in terms of two domains.The core of the larger domain is represented by a seven-stranded and a four-stranded antiparallel β sheet, structurally joined by the S4 strand (110–120) that is part of both sheets. The β sheets core region is flanked by H1 (and H0 in PLP-free *e*PNPOqm) on one side and H2 on the opposite side. The smaller domain packs adjacent to the β-barrel core and helix H2 and is made up by the three remaining α helices, H3, H4, and H5 (154–166) (Fig. 3*A*).

In the PLP-*e*PNPOqm model, the subunit A backbone displays a major break of 49 residues (comprising residues 127–175), corresponding to the *e*PNPO smaller domain comprising the H3, H4, and H5 helices, whereas only a minor break of three residues is present in subunit B (residues 167–169), which could not be modelled because of the lack of information from the F_O_ – F_C_ map (Fig. 3*B*).

No PLP is present at the active site of either structures (Fig. 3, *A* and *B*), although PLP-*e*PNPOqm was crystallized in the presence of excess PLP (see Experimental procedures section for details); this is evidently a consequence of the engineered active site variants (Fig. 3, *C* and *D*).

In contrast to the symmetrical *e*PNPOqm structure, the PLP-*e*PNPOqm is the first ever characterized structure of the enzyme crystallized in a P6_1_ space group, in which the dimer is asymmetric. This structural discrepancy resides in the major chain break contained in subunit A, comprising a portion of H3 (residues 123–130) and the whole H4 and H5 (residues 135–143 and 153–166, respectively) regions.

**Figure 4:**
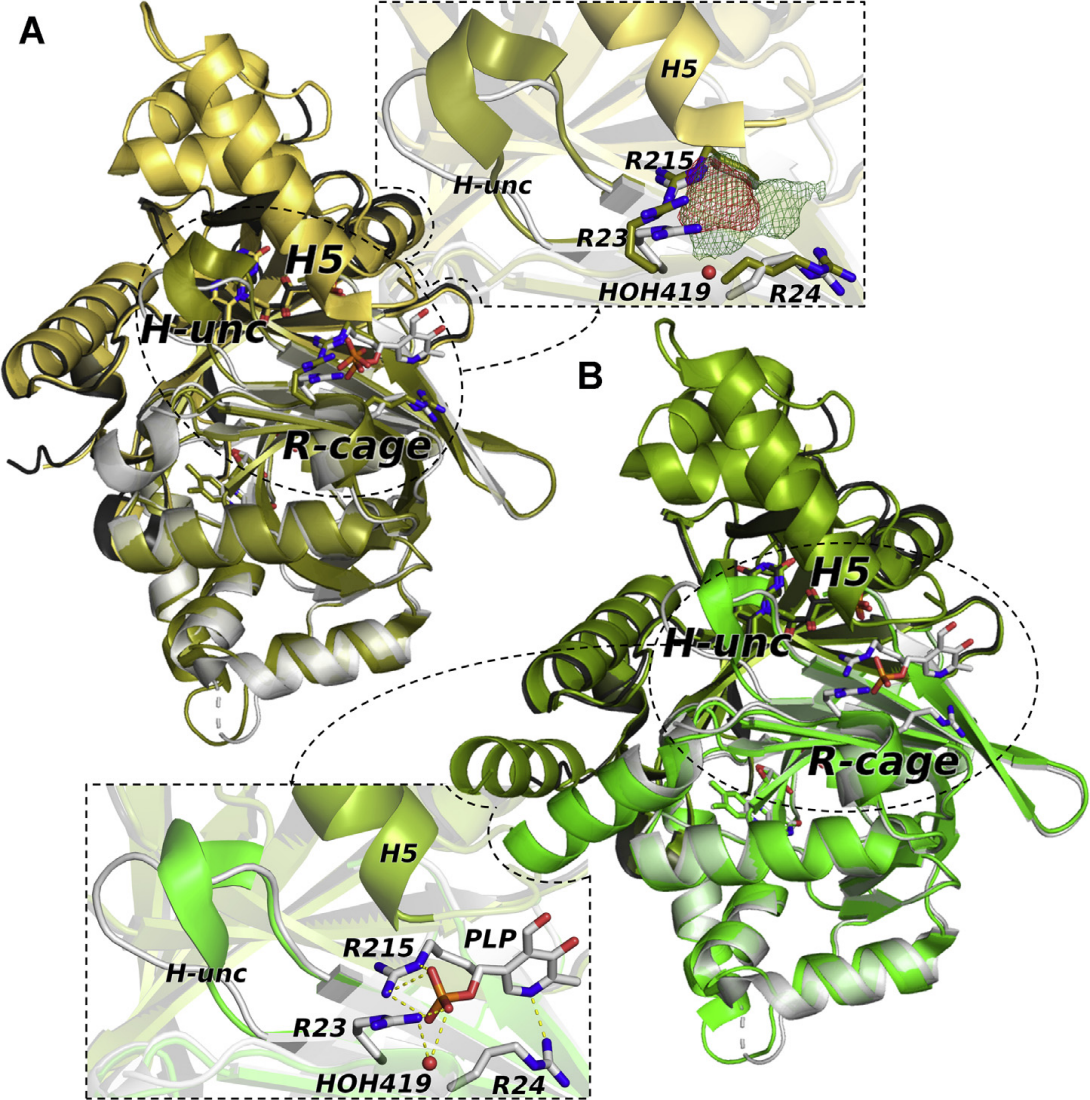
**The Arg cage.***A*, superimposition between the structures of wildtype *E. coli* pyridoxine 5’-phosphate oxidase (*gold*) (Protein Data Bank code: 1G79) and pyridoxal 5’-phosphate (PLP)-bound *e*PNPOqm (*gray*), and blow-up showing the Fo–Fc omit maps (*green* for PLP, contained in PLP-*e*PNPOqm, and *red* for phosphate, contained in the 1G79 structure). By contouring at 2.5 σ, a significant difference in steric hindrance is visible, revealing the presence of a bulkier ligand with respect to a phosphate ion. The side chains of arginine residues lining the cage are represented as *sticks*. *B*, superimposition between the structures of *e*PNPOqm in the free (*green*) and PLP-bound (*gray*) forms and blow-up showing that PLP is seized in the superficial cleft mainly *via* electrostatic interactions, mediated by three arginine residues. A water molecule (*red sphere*) bridges Arg23 and the PLP phosphate group through a hydrogen bonds network. PLP and the side chains of arginine residues lining the cage are represented as *sticks*.

The disorder of these elements in subunit A seems to occur as a consequence of PLP binding in a surface cleft, placed in part at the interface between subunits A and B, mainly circumscribed by three arginine residues (Arg23, Arg24, Arg215) belonging to subunit B and acting as an electrostatic “Arg cage” for PLP. The three Arg residues efficiently trap PLP in the cavity (Fig. 4, *A* and *B*). The binding driving force is represented by the phosphate group, as often occurs in PLP-binding proteins (11), which is capable of establishing polar interactions with the amino groups of the side chains and backbone of Arg23, Arg215, and Arg24, respectively.

In addition, a small network of hydrogen bonds takes place between Arg23 and the PLP phosphate group by means of a bridging water molecule, HOH419, whose role is apparently to contribute to stabilizing PLP lodging in the cavity; this effect is enhanced by the pyridine ring, able to coordinate Arg24 and Arg215 *via* the deprotonated nitrogen atom. This Arg cage was previously identified by Safo and collaborators (8) (PDB code: 1G76, 1G77, 1G78, 1G79) as a binding site for a unique phosphate ion; the PLP phosphate group and this phosphate ion appear to bind in a very similar fashion, as only a slight displacement of Arg23 is detectable in the same binding site. The comparison between the corresponding Fo–Fc omit maps (contoured at 2.5σ) (Fig. 4*A*; Fig. S4) allows us to exclude the presence of a phosphate ion rather than PLP in this cavity of subunit B of the PLP-*e*PNPOqm structure.

### Conformational change induced by PLP binding

**Figure 5:**
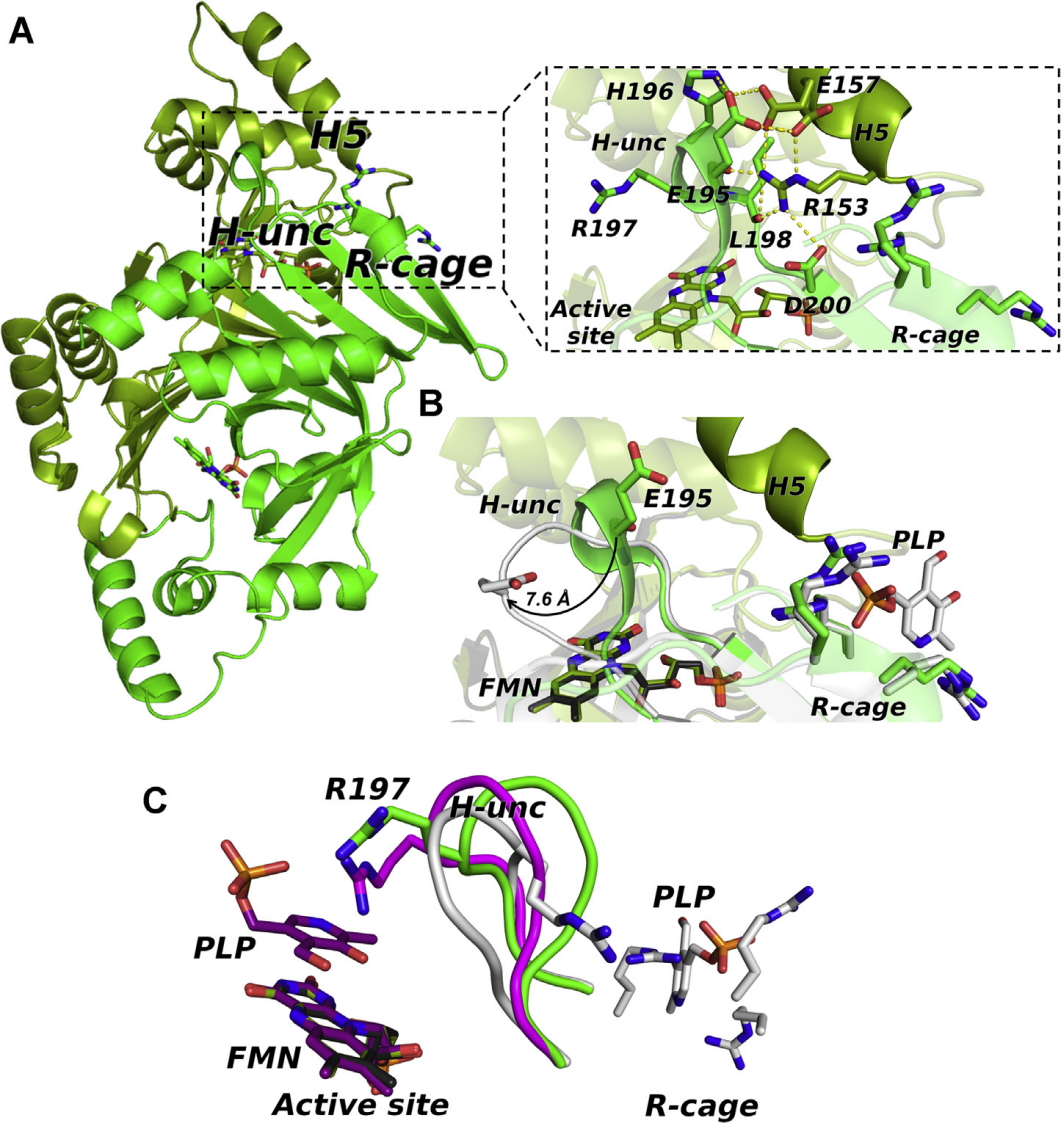
Conformational change induced by PLP binding to PNPOqm. *A*, overall fold of *e*PNPOqm (without pyridoxal 5’-phosphate [PLP]) crystallized in the P3_1_ 2 1 space group and blow-up of the anchoring region in *e*PNPOqm. The second subunit of the dimer is generated by the crystallographic 2-fold axis. The two symmetric subunits of the dimer are colored in *light* (subunit A) and *dark* (subunit B) *green*; key residues are shown as *sticks*. *B,* detail of superimposed *e*PNPOqm (*green*) and PLP-*e*PNPOqm (*gray*) anchoring regions. Upon PLP binding, the 192 to 200 H-unc structural element is displaced of 7.6 Å. *C,* structural superimposition of the 192 to 200 H-unc section**,** shown as a *ribbon*, in wildtype *E. coli* pyridoxine 5’-phosphate oxidase (1G76; in *purple*), *e*PNPOqm (in *green*), and PLP-*e*PNPOqm (in *gray*) (the structures are rotated about 30° clockwise with respect to the position in *A* and *B*). Upon PLP binding, Arg197 shifts and its side chain rotates about 180° around its Cα.

The superimposition of PLP-*e*PNPOqm and *e*PNPOqm revealed important differences between the two structures, highlighting the structural change induced by PLP binding to the allosteric site. Indeed, PLP binding causes the dismantling of a specific pattern of hydrogen bonds and electrostatic interactions anchoring H5 of subunit A to a short neighboring protein segment made of residues 192 to 200 of subunit B (“H-unc”; Fig. 5, *A* and *B*).

In this structure of *e*PNPOqm without PLP, the interactions between two key residues of H5 (subunit A; Arg153 and Glu157) and residues Glu195, Leu198, and Asp200 placed on the 192 to 200 helix (subunit B) are particularly important to maintain these structural elements linked together and correctly folded (Fig. 5*A*). Binding of PLP to subunit A of *e*PNPOqm causes a displacement of the H-unc structural element (C_α_ of Glu195 moves 7.6 Å apart from its previous position), thereby disrupting the interactions of this structural element with H5 of subunit A, which loses its structure together with H3 and H4 (Fig. 5*B*).

The displacement of H-unc upon PLP binding causes also the movement of Arg197 (placed on H-unc), which, in the wildtype *e*PNPO structure (PDB code: 1G76), contributes to the PLP binding to the catalytic site, establishing electrostatic interactions with both its aldehyde and hydroxyl groups. As shown in Figure 5*C*, in the *e*PNPOqm structure, the Arg197 displays a position similar to that of the wildtype protein. Upon PLP binding to the Arg-cage site, Arg197 shifts and its side chain rotates about 180° around its Cα with respect to its original position (Fig. 5*C*). The large conformational change of the 190 to 200 segment, and in particular the displacement of Arg197, alters the structure of the active site and therefore the affinity for both PLP and substrate, and as a consequence reduces the catalytic activity of the enzyme.

### Proving the identity of the allosteric PLP-binding site revealed by the present crystallographic data

Since the amino acid residues Arg23, Arg24, and Arg215 are crucial for the interaction with PLP in the PLP-*e*PNPOqm crystal structure, single variant forms (R23L and R215L) as well as two double (R23L/R24L and R23L/R215L) and a triple (R23L/R24L/R215L) *e*PNPO variants were produced through site-directed mutagenesis and characterized. All variants showed far-UV CD spectra superimposable to that of the wildtype *e*PNPO (Fig. S2), indicating that the variations did not affect the secondary structure of the protein. Data obtained from DSF measurements showed that all variants are quite similar to the wildtype enzyme with respect to thermal stability (Fig. S3*B*) (Table 1). With these variants, enzyme activity measurements yielded *K*_M_ and *k*_CAT_ values that, in some cases, are different with respect to those of wildtype *e*PNPO (Table 1); however, they testify to substantial preservation of the enzyme functional properties.

PLP binding was analyzed through the same spectrofluorometric method described above (Fig. S5*B*). Noticeably, the PLP binding properties of all variants were found to be substantially altered, as shown by K_D_ values that in some cases are 7- to 10-fold higher than that of the wildtype enzyme (Table 1). When *e*PNPO variants were incubated with PLP and passed through an SEC column, a considerably low percentage of PLP was retained: only 18% to 19% (with respect to protein subunits) in the case of the R23L/R215L and R23L/R24L/R215L variants, in comparison with the 70% value obtained with wildtype *e*PNPO (Table 1).

### Allosteric properties of Arg-cage variants

**Figure 6:**
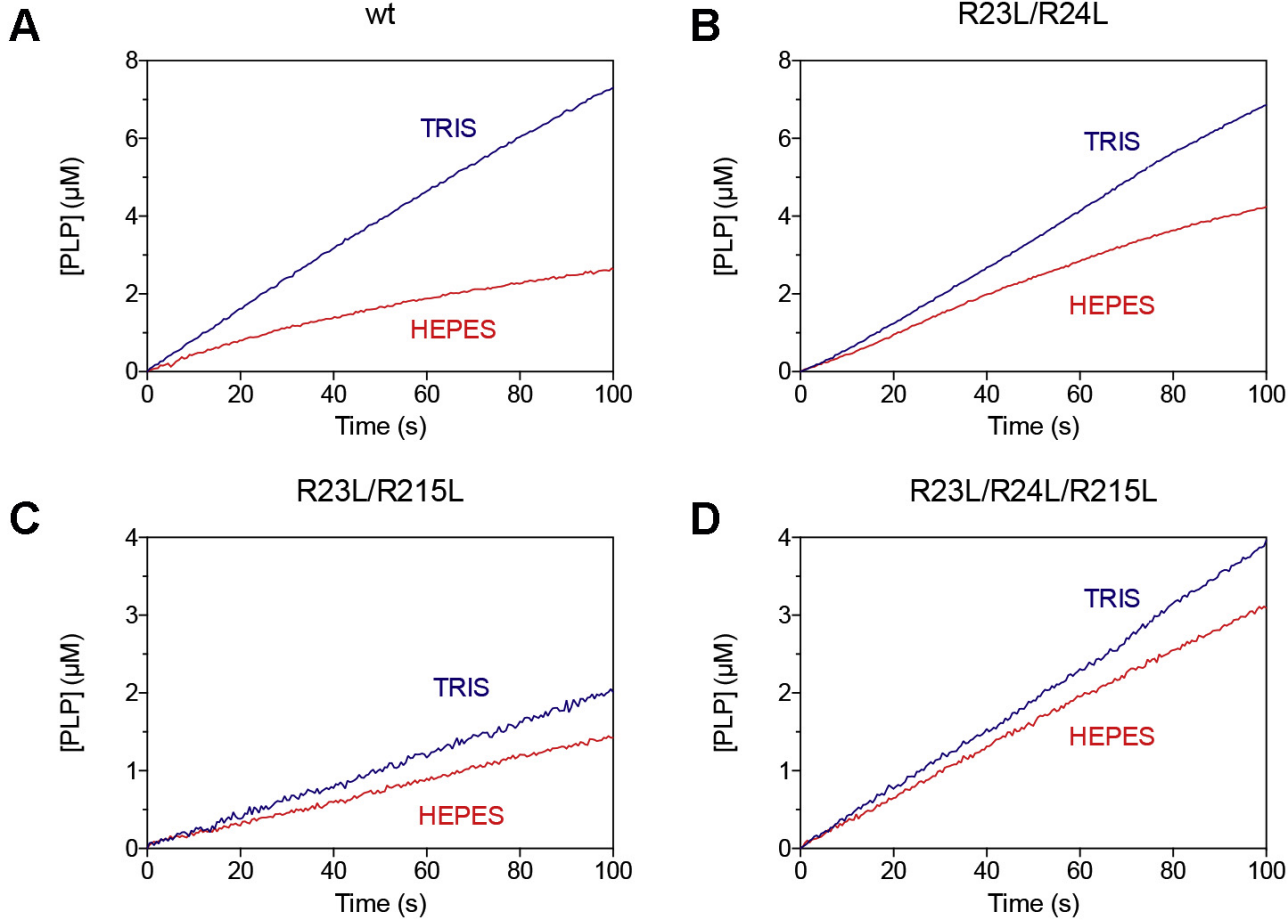
Effect of Arg-cage variations on allosteric inhibition. **Comparison of kinetics carried out in 50 mM Tris-HCl and 50 mM Na-Hepes buffers at pH7.6, obtained using 0.5 μM enzyme(*A*, WT; *B*, R23L/R24L; *C*, R23L/R215L; *D*, R23L/R24L/R215L)and 15 μM pyridoxine 5’-phosphate**.

As mentioned above, kinetic measurements of PNP oxidation into PLP catalyzed by PNPO are usually carried out in Tris buffer to prevent PLP accumulation and enzyme inhibition (9). Studies carried out by our group showed that, when the reaction is carried out in Hepes buffer, a complex kinetics of PLP formation is observed as a consequence of PLP allosteric inhibition (5). Initially, upon mixing enzyme with PNP, the velocity of product formation decreases, resulting in a deceleration phase caused by the accumulation of PLP in the solvent, binding of PLP at the allosteric site, and the consequent onset of enzyme inhibition (Fig. 6*A*). The first deceleration phase is followed by a slower, approximately linear production of PLP. This complex inhibition behavior has been interpreted according to the model shown in Figure 2*A*, which is that of an allosteric linear mixed-type inhibition. Kinetics of PNP oxidation catalyzed by wildtype and Arg-cage variants were examined in Tris and Hepes buffers, and compared. As shown in Figure 6, in Tris buffer all variant enzymes behave very similarly to the wildtype enzyme, showing a linear rate of product formation, followed by a gradual decrease of the reaction rate only when substrate depletion becomes significant. On the other hand, in Hepes buffer PLP inhibition is clearly attenuated in all variant forms with respect to wildtype *e*PNPO. In particular, with the R23L/R215L and R23L/R24L/R215L variants, PLP production in Hepes buffer is almost linear (Fig. 6, *C* and *D*, respectively).

**Figure 7:**
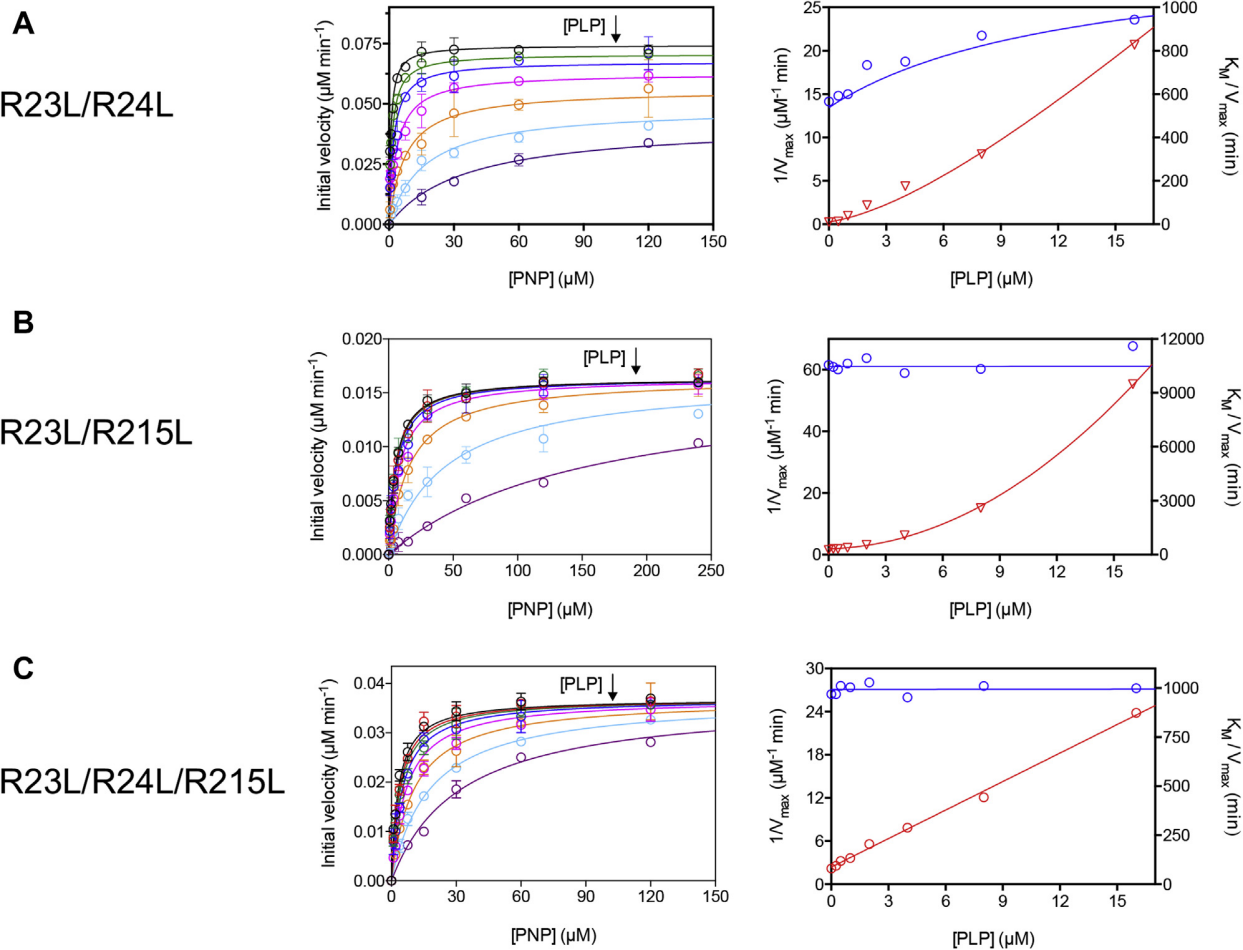
Kinetic analysis of PLP inhibition with Arg-cage variants. **Characterization of pyridoxal 5’-phosphate (PLP) inhibition of *E. coli* pyridoxine 5’-phosphate oxidase R23L/R24L (*A*), R23L/R215L (*B*), and R23L/R24L/R215L (*C*) variants.** The initial velocity of the reaction was measured with 0.5 μM enzyme (protein subunit concentration), varying pyridoxine 5’-phosphate (PNP) concentration (between 0 and 240 μM) while keeping exogenous PLP fixed at different concentrations (0, 0.25, 0.5, 1, 2, 4, 8, and 16 μM). Initial velocity measurements shown in the *left panels* are the average ± standard deviation of three independent measurements. Saturation curves were fitted to the Michaelis–Menten equation, obtaining apparent *V*_MAX_ and *K*_M_ values as a function of PLP concentration. These are represented as 1/*V*_MAX_ (*blue symbols*) and *K*_M_/*V*_MAX_ (*red symbols*) in the *right panels*, in order to facilitate the interpretation of the PLP inhibition mechanism from the shape of graphs, which correspond to the classic secondary plots of the Y-intercept and slope derived from Lineweaver–Burk plots. As detailed in the Experimental Procedures section, initial velocity data and 1/*V*_MAX_ and *K*_M_/*V*_MAX_ values of each data set were globally fitted to Equations (3), (4), (5), obtaining the continuous lines shown in the graphs and the parameter values shown in Table 3.

A thorough analysis of PLP inhibition with the R23L/R24L, R23L/R215L and R23L/R24L/R215L variants was performed in Hepes buffer. The initial velocity of PLP production was measured as a function of PNP concentration at different fixed PLP concentrations (from 0.25 to 16 μM). Initial velocity data obtained with all variant forms were fitted to the Michaelis–Menten equation in order to obtain apparent *V*_MAX_ and *K*_M_ values as a function of PLP concentration. With wildtype *e*PNPO (5), PLP affects both apparent *V*_MAX_ and *K*_M_. Plots of 1/*V*_MAX_ and *K*_M_/*V*_MAX_ as a function of PLP concentration are linear, indicating a linear mixed-type inhibition, in which the enzyme activity is completely abolished at infinite PLP concentration (*i.e.*, the ternary substrate–enzyme–product complex, PES, is completely inactive; Fig. 2*A*). It is striking that, with the R23L/R24L *e*PNPO variant, 1/*V*_MAX_ shows a hyperbolic increase, whereas *K*_M_/*V*_MAX_ increases with a parabolic shape (Fig. 7*A*). This behavior indicates that an infinite PLP concentration leads to a partial inhibition, in which the PES complex is catalytically competent but has a lower activity as compared with the ES complex; moreover, the peculiar parabolic shape of *K*_M_/*V*_MAX_ indicates that PLP inhibits the enzyme by binding at two distinct sites, possibly at the active site and at an allosteric site, causing a parabolic inhibition. A still different behavior is observed with the R23L/R215L variant. In this case, 1/*V*_MAX_ is not affected by PLP concentration, suggesting that the PES complex does not form; however, *K*_M_/*V*_MAX_ shows a marked parabolic increase (Fig. 7*B*). Finally, the R23L/R24L/R215L triple variant shows a pure competitive inhibition behavior with respect to PLP, in which 1/*V*_MAX_ is constant and *K*_M_/*V*_MAX_ has an increasing linear dependence on PLP concentration (Fig. 7*C*), indicating that PLP inhibits the enzyme activity by binding only at the active site, competing with the substrate PNP.

**Figure 8:**
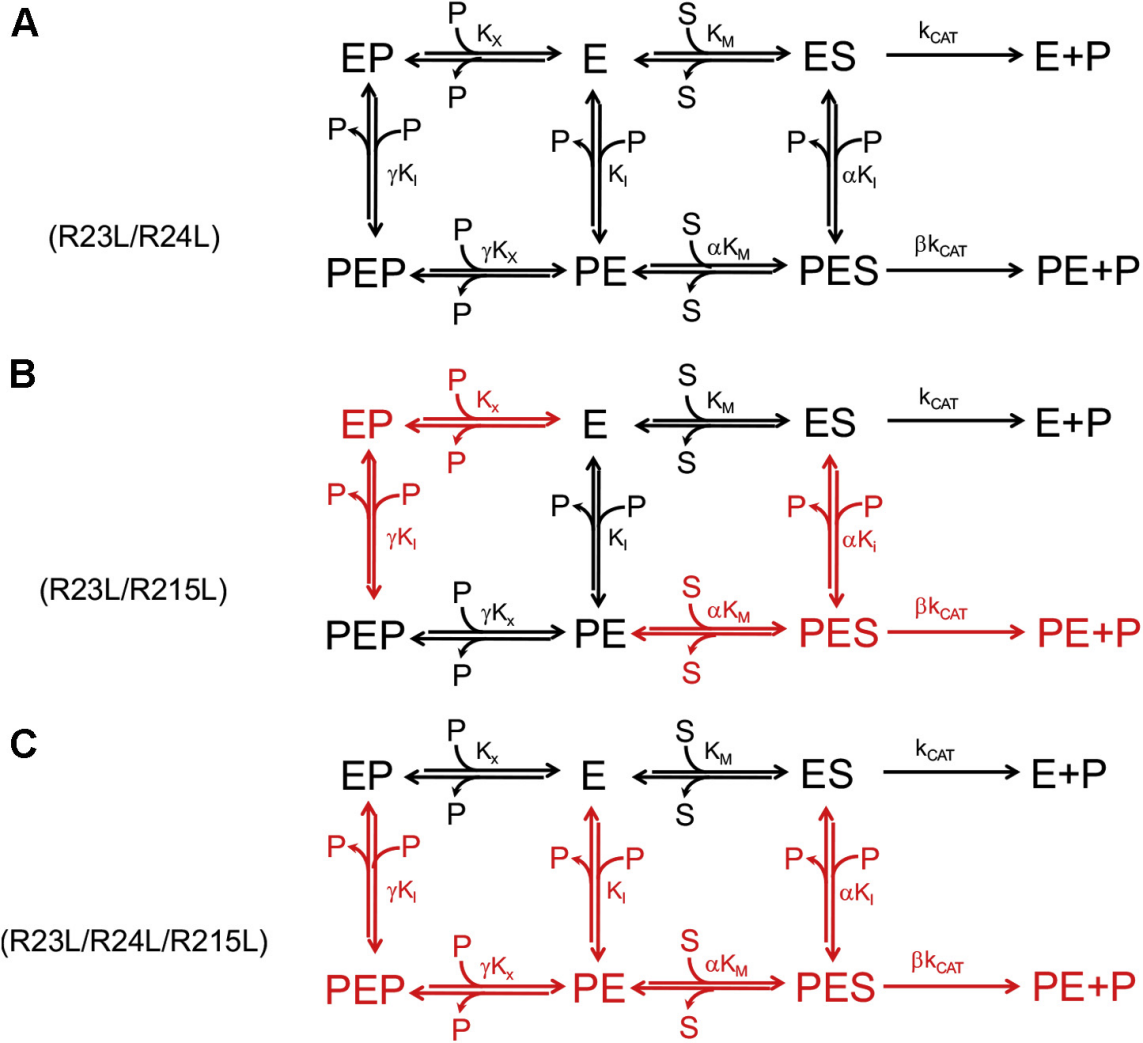
**Steady-state kinetic models describing inhibition of *Escherichia coli* pyridoxine 5’-phosphate oxidase Arg-cage variants by pyridoxal 5’-phosphate.***A*, general inhibition model in which pyridoxal 5’-phosphate (PLP) (P) can bind to free enzyme (E) at either the active site (forming the EP complex) or the allosteric site (forming the PE complex). Then, a second PLP molecule can bind at the other available site, forming the ternary PEP complex. Binding of PLP at a site influences binding at the other site, so that the dissociation constants of PLP binding equilibria at the active site (K_X_) and at the allosteric site (K_I_) are modified by a γ coefficient. Binding of substrate at the active site and binding of PLP at the allosteric site influence each other, so that the related dissociation constants (*K*_M_ and K_I_) are increased by an α coefficient. The enzyme–substrate complex (ES) can either be transformed into PLP product and free enzyme (with a rate constant corresponding to *k*_CAT_) or bind PLP at the allosteric site, forming the PLP–enzyme–substrate ternary complex (PES), which can be transformed into PLP plus PE, although with a *k*_CAT_ that is reduced by a β coefficient. *B*, in this model some of the binding equilibria present in the general inhibition model (*A*) do not take place and are therefore shown in red. Once the free enzyme has bound the substrate at the active site, forming the ES complex, it is not capable of binding PLP at the allosteric site. Conversely, once the free enzyme has bound PLP at the allosteric site, forming the PE complex, it is not capable of binding the substrate at the active site. As a consequence, the PES ternary complex is not formed. The PEP ternary complex can be formed, but only upon binding of PLP to the active site of the PE complex. In this respect, this model is similar to that used to explain the PLP inhibition behavior of the human pyridoxine 5’-phosphate oxidase (6). *C*, also, in this model some of the binding equilibria present in the general inhibition model (*A*) do not take place and are therefore shown in *red*. This is a simple competitive inhibition model, in which the enzyme is not capable of binding PLP at the allosteric site.

In order to analyze the above-mentioned kinetic data, a general inhibition model was considered (Fig. 8*A*), in which PLP binds at both the active and allosteric sites. Binding of PLP at the allosteric site and binding of substrate at the active site influence each other, increasing *K*_M_ and K_I_ by an α coefficient. Analogously, binding of PLP at the active site and at the allosteric site increases the dissociation constants of the respective equilibria by a γ coefficient. In this model, although PLP binding at the active site obviously leads to complete inactivation, catalysis is only partially impaired when PLP binds at the allosteric site (*k*_CAT_ is multiplied by a β coefficient, whose value ranges from 0 to 1). In this model, all equilibria are assumed to be rapidly established upon mixing of the enzyme with substrate and PLP, and conversion of substrate into product to be relatively slow; indeed, *k*_CAT_ varies from 0.05 to 0.25 s^−1^ in all variant and wildtype enzymes (Table 1), whereas PLP binding is much faster (*k*_ON_ = 11.3 μM^−1^s^−1^ and *k*_OFF_ = 3.2 s^−1^, (5)). Therefore, the dissociation constant of the substrate binding equilibrium may be assumed to correspond to the Michaelis–Menten constant (*K*_M_) derived from steady-state kinetic measurements. Equations describing the dependence of initial velocity, 1/*V*_MAX_, and *K*_M_/*V*_MAX_ on substrate and PLP concentration were derived on the basis of these assumptions (Equations (3), (4), (5)). Using these equations, initial velocity data as a function of substrate concentration, obtained at all different fixed PLP concentrations, were globally fit together with the previously obtained values of apparent 1/*V*_MAX_ and *K*_M_/*V*_MAX_ as a function of PLP concentration.

Initially, global fitting of data acquired with the R23L/R24L variant was carried out by letting all parameters free to vary from zero to 10^10^, except β, which was constrained between 0 and 1. This approach resulted in a good fit, in which theoretical curves matched very well the experimental points (Fig. 7*A*) and all parameters were determined, but some parameters (K_I_, K_X_, and γK_X_) were “ambiguous,” *i.e.*, were determined with large standard errors. On the other hand, standard errors for the other parameters were acceptable: *V*_MAX_ = 0.074 ± 0.001 μM s^−1^, *K*_M_ = 0.80 ± 0.08 μM, αK_I_ = 5.39 ± 1.14 μM, and β = 0.42 ± 0.05. This situation is typically caused by a large number of unconstrained parameters. By fixing all “unambiguous” parameters (*V*_MAX_, *K*_M_, αK_I_, and β at the values obtained in the previous fitting procedure), all other parameters were determined, with values equal to those returned by the previous fitting but with acceptable standard errors (Table 3), showing that the model depicted in Figure 8*A* adequately describes the results obtained with the R23L/R24L variant.

**Table 2:** Crystal parameters, data collection, and refinement statistics

Parameters and refinement statistics	*e*PNPOqm (K72I/Y129F/R133L/H199A)	PLP-*e*PNPOqm (K72I/Y129F/R133L/H199A)
PDB code	6YLZ	6YMH
Space group	P3_1_ 2 1	P6_1_
Cell constants a, b, c, α, ɓ, ɣ	63.78 Å 63.78 Å 124.73 Å 90.00° 90.00° 120.00°	54.16 Å 54.16 Å 271.97 Å 90.00° 90.00° 120.00°
Asymmetric unit (residues/chain)	Monomer (7/A - 218/A)	Dimer (17/A–126/A, 176/A–218/A; 14/B–166/B, 170/B–218/B)
Resolution range (Å)	55.232–1.558 (1.585–1.558)	46.907–2.417 (2.459–2.417)
Unique reflections	41,877 (2040)	17,206 (898)
Completeness (%)	98.0 (98.0)	100.0 (100.0)
Redundancy	19.7 (11.9)	20.1 (17.2)
R_merge_	0.13 (2.72)	0.21 (3.55)
CC(1/2) (%)	99.9 (44.4)	99.9 (39.2)
I/σ(I)	13.6 (0.9)	11.9 (0.9)
High-resolution limit having I/σ(I) higher than 2 (Å)	1.7	2.6
Resolution range (Å) for refinement	55.232–1.558 (1.599–1.558)	46.907–2.417 (2.480–2.417)
R_work_ (%)	17.06 (36.50)	18.97 (37.20)
R_free_ (%)	20.22 (36.30)	25.88 (42.60)
Rms (bond_angle) (°)	1.721	1.784
Rms (bond_length) (Å)	0.013	0.014
Wilson B-factor (Å^2^)	23.6	58.5
Residues in favored regions of Ramachandran plot (%)	97.0	97.0
Residue in allowed regions of Ramachandran plot (%)	3.0	2.0
Ramachandran outliers (%)	0.0	1.0
Ligands (Real Space Correlation Coefficient, RSCC)	-	PLP (0.89)

Values for the high-resolution shell are listed in parentheses.

**Table 3:** Parameters obtained from global fitting of PLP inhibition data obtained with *e*PNPO R23L/R24L, R23L/R215L, and R23L/R24L/R215L variants

Parameters	aWT	R23L/R24L	R23L/R215L	R23L/R24L/R215L
*V*_MAX_ (μM s^−1^)	0.135 ± 0.005	0.074 ± 0.001	0.016 ± 0.001	0.037 ± 0.001
*K*_M_ (μM)	0.9 ± 0.55	0.80 ± 0.08	5.40 ± 0.22	3.19 ± 0.09
β	-	0.42 ± 0.05	-	-
K_I_ (μM)	0.59 ± 0.7	0.82 ± 0.02	13.28 ± 2.45	-
αK_I_ (μM)	7.13 ± 0.52	5.39 ± 1.14	-	-
K_X_ (μM)	-	6.58 ± 0.62	-	1.78 ± 0.06
γK_X_ (μM)	-	2.05 ± 0.06	0.73 ± 0.14	-

Missing parameters do not occur in the models used to fit experimental data.

a Determined according to Figure 2*A* by Barile *et al.* (5).

With the R23L/R215L variant, the same initial fitting strategy was adopted. Also in this case, a good fit was obtained and all parameters were determined; however, some parameters (β, K_I_, αK_I_, K_X_, and γK_X_) had large standard errors, whereas *V*_MAX_ was equal to 0.016 ± 0.001 μM s^−1^ and *K*_M_ was 5.40 ± 0.22 μM. Noticeably, the determined value for β was close to zero, whereas K_X_ and αK_I_ values were very large (≥10^3^). The very large α value strongly indicates that the PES complex is not formed (Fig. 8*B*), and this would explain why the β value is close to zero. As a matter of fact, fixing the β value either to zero or to 1 gave equally good fits and parameters with the same values determined in the previous fitting, supporting this hypothesis. Therefore, a second fitting procedure was performed, in which β was fixed to zero, K_X_ and αK_I_ were fixed to a large value (10^4^), and *V*_MAX_ and *K*_M_ were fixed to the values obtained in the previous fitting procedure. A good fit was obtained (Fig. 7*B*) with K_I_ = 13.28 ± 2.45 μM and γK_X_ = 0.73 ± 0.14 μM, which correspond to the values returned by the previous fitting.

As argued above, in the case of the R23L/R24L/R215K variant, it is clear that PLP acts as a pure competitive inhibitor. Therefore, the global fitting of inhibition data obtained with this variant was carried out according to Figure 8*C*, by fixing K_I_, αK_I_, and γK_X_ to a large value (10^4^) and β to zero. In this way, a good fit was obtained with values of *V*_MAX_ = 0.037 ± 0.001 μM s^−1^, *K*_M_ = 3.19 ± 0.09 μM, and K_X_ = 1.78 ± 0.06 μM (Fig. 7*C*).

**Figure 9:**
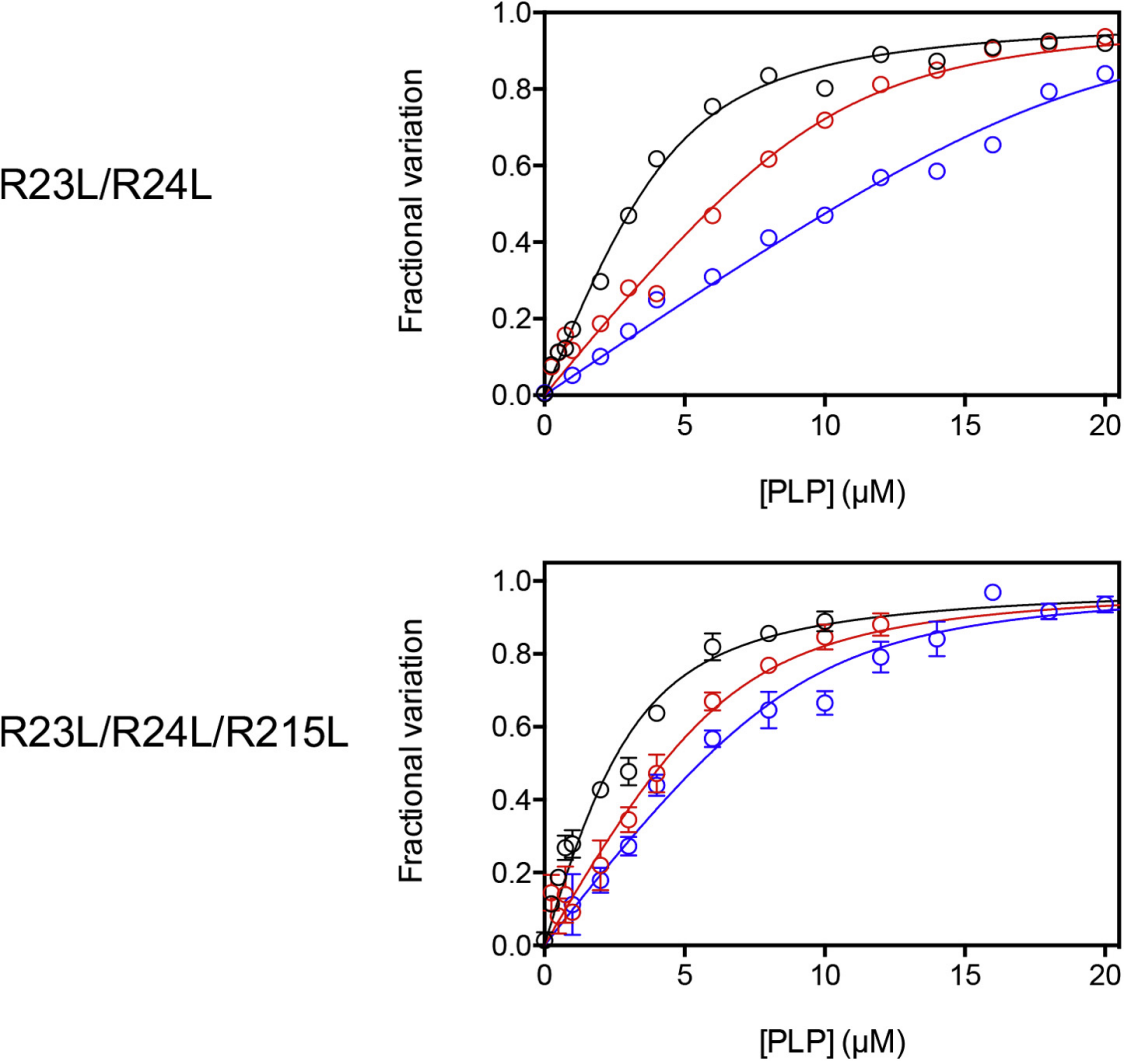
**Analysis of pyridoxal 5’-phosphate (PLP)-binding stoichiometry.** Fluorescence emission spectra (from 470 to 570 nm) of *E. coli* pyridoxine 5’-phosphate oxidase were acquired upon excitation of the FMN cofactor at 450 nm, in the presence of increasing PLP concentrations. Three different protein subunit concentrations were used: 2 (*black symbols*), 4 (*red symbols*), and 8 μM (*blue symbols*). Fluorescence change, expressed as fractional variation as a function of PLP concentration, was fitted to a quadratic equation Equation 2 describing binding of a ligand to equivalent sites. In the fitting procedure, dissociation constants (K_D_) were fixed at the values determined from experiments carried out with the same variants using smaller protein concentrations (Table 1), whereas the protein subunit concentration was let free to vary between zero and infinite. The fitting returned the continuous lines shown in the figure and the following estimated values of protein subunit concentrations: 4.44 ± 0.23, 10.26 ± 0.89, and 19.17 ± 0.53 μM for the R23L/R24L variant; 2.76 ± 0.36, 6.38 ± 0.47 and 9.04 ± 0.56 μM for the R23L/R24L/R215L variant.

In order to verify the indications concerning PLP binding to *e*PNPO emerged from the fitting of inhibition data, the PLP binding stoichiometry was measured, using the previously described fluorimetric method, with the R23L/R24L and the R23L/R24L/R215L variants. Titration of concentrated *e*PNPO solutions (2, 4, and 8 μM) with PLP clearly indicated a stoichiometry of two molecules of PLP per protein subunit with R23L/R24L *e*PNPO and of one molecule of PLP per protein subunit with R23L/R24L/R215L *e*PNPO (Fig. 9).

## Discussion

Our site-directed mutagenesis studies showed that the secondary site revealed by previous crystallographic investigations (8) does not correspond to the PLP tight binding site, since all variants (K145A/F177A, N84A/K145A/F177A and N84W/K145A/F177A) have substantially unaltered K_D_ values for PLP binding and full capability to retain PLP after incubation with this vitamer and subsequent SEC. Conversely, the outcomes of the variations that target the Arg cage identified in our crystallographic studies (R23L, R215L, R23L/R24L, R23L/R215L and R23L/R24L/R215L) clearly indicate a perturbation of PLP binding, testified by a 10-fold increase of K_D_ and a substantial decrease of PLP retention. Disruption of the PLP tight binding site may be expected to result in a more drastic effect, such as the complete abolishment of PLP binding. However, when interpreting these results, the hypothesis that PLP may also bind to another site, such as the active site, should be taken into consideration, and actually, it offers a very plausible explanation, as clearly indicated by inhibition kinetics and PLP binding stoichiometry studies discussed below. Therefore, our results clearly demonstrate that the Arg cage does correspond to the PLP tight binding allosteric site.

In wildtype *e*PNPO, the enzyme activity is regulated by a strong allosteric coupling between two binding events: PLP binding at the allosteric site and substrate binding at the active site. The reciprocal influence of these events is visible in the increase of the respective dissociation constants (by α factor of α = 12) and in the lack of catalytic activity of the PLP–enzyme–PNP ternary complex (PES in Fig. 2*A*). Clearly, this is a consequence of structural perturbations induced on the protein by PLP and PNP. The effect of PLP binding at the allosteric site is evident in the crystal structure of PLP-*e*PNPOqm. PLP binding at the Arg cage results in the disorder of a large structural region made by a portion of helix H3 (residues 123–130) and the whole H4 and H5 helices (residues 135–143 and 153–166, respectively); moreover, it uncoils the short H-unc helix (192–200 residues), which is a very important structural element of the active site. In this rearrangement, the Arg197 side chain is moved away from FMN and rotated about 180° around its Cα with respect to that of *e*PNPOqm, so that it interacts with the peptide carbonyl oxygens of the N-terminal Thr18, Lys19, and Gly20 residues. Arg197, which is highly conserved in PNPO from diverse sources, is involved in substrate binding and orientation for a correct and efficient catalysis (8). The side chain of Arg197 helps position the PNP pyridine ring parallel to the FMN isoalloxazine ring and interacts with its phosphate moiety, so that the substrate is correctly oriented for an efficient and stereospecific transfer of the C4’ pro-R hydrogen to FMN. In *E. coli* PNPO, the R197E variation results in an 8000-fold increase in *K*_M_ and a 16-fold decrease in *k*_CAT_ (12). The His199 residue is also involved in substrate binding by interacting with the phosphate moiety (8). For all these reasons, the structural rearrangement observed in PLP-*e*PNPOqm explains perfectly well the allosteric effect of PLP on PNP binding at the active site (increase of *K*_M_) and also the catalytic incompetence of the PES complex. By the reverse reasoning, it is expected that PNP binding at the active site, and the consequent interaction of the substrate with Arg197 and H199 of the short 192 to 200 helix, may affect PLP binding at the allosteric site. Nonetheless, it should be pointed out here that the structure of the PLP-*e*PNPOqm dimer is asymmetric in that PLP is bound only to one subunit and the discussed structural changes concerning helices H3, H4, and H5 and the 192 to 200 short helix are limited to that subunit. From the PLP-*e*PNPOqm structure, it is not clear why binding of PLP to both allosteric sites of the dimer is prevented. However, in solution the stoichiometry of PLP binding to the allosteric site of wildtype *e*PNPO is actually of one PLP molecule per protein dimer (5), indicating that the allosteric effect of PLP binding to one subunit extends to the other subunit, preventing the binding of a second PLP molecule. On the other hand, with the *e*PNPO quadruple variant (having an impaired active site), this stoichiometry ratio is changed to two PLP molecules per dimer (5), indicating that the substantial alteration of the active site caused by the variations have somewhat mitigated the allosteric effect of PLP binding at the allosteric site. Hence, the asymmetry of the PLP-*e*PNPOqm structure may result from the crystalline state of the protein that has populated that particular asymmetric conformation.

Kinetic inhibition studies provide additional insight into the Arg-cage variants. These variations have the effect to uncouple the allosteric site and the active site to different extents. In contrast to wildtype *e*PNPO, the R23L/R24L variant is capable of binding PLP at both the allosteric site and the active site (Fig. 8*A*), as also demonstrated by a PLP binding stoichiometry ratio of four PLP molecules per enzyme dimer (Fig. 9). This is because the allosteric coupling between the two sites is considerably reduced (α = 6.6), as also seen in the fact that the PES ternary complex maintains part of the catalytic activity (α = 0.42); PLP binding at the allosteric site does affect the active site structure but not as much as in wildtype *e*PNPO. On the other hand, PLP binding at the active site is positively influenced by PLP binding at the allosteric site (and this is a reciprocal effect), as indicated by a factor γ = 0.31. Of interest, we observed a similar behavior with human PNPO (6) (Fig. 2*B*), although in this case γ was equal to 6.6. A different behavior is observed with the R23L/R215L variant. In this case, different outcomes result from binding of either PLP or substrate to the free enzyme. Just like wildtype *e*PNPO, the free enzyme can only bind PLP at the allosteric site; however, once the PE complex is formed (Fig. 8*B*), this cannot bind the substrate but it is capable of binding a second PLP molecule at the active site, forming the inactive PEP complex. On the other hand, once the free enzyme has bound the substrate at the active site, then it is not capable of binding PLP at the allosteric site, but it is perfectly able to catalyze the conversion of the substrate into product. Eventually, the replacement of all three arginines of the Arg cage with Leu residues completely abolishes PLP binding at the allosteric site, or at least any allosteric effect of PLP binding, so that PLP can only bind at the active site. This is clearly seen from the pure competitive inhibition observed with the R23L/R24L/R215L variant (Fig. 8*C*), whose analysis returns a K_X_ value (1.78 μM) that is quite similar to the K_D_ measured in the fluorimetric studies of PLP binding to the free enzyme (1.03 μM). In this respect, the substantial increase of K_D_ for PLP binding and the decrease of PLP retention observed with the Arg-cage variants (in particular with R23L/R215 and R23L/R24L/R215L), as opposed to the expected complete abolishment of PLP binding, are explained by the fact that, in all these variants, PLP binds at the active site with appreciable affinity. The allosteric effect of PLP is either strongly decreased or abolished with R23L/R215 and R23L/R24L/R215L variants, respectively, as evident from a constant *V*_MAX_ as a function of PLP concentration (Fig. 7), although in the former PLP binds at the allosteric site (but the PES complex cannot be formed). Comparison of PLP formation kinetics obtained in Tris and Hepes buffer (Fig. 6) also indicates the loss of the allosteric effect. With both R23L/R215 and R23L/R24L/R215L variants, kinetics in Hepes is almost linear, as opposed to that observed with wildtype and R23L/R24L *e*PNPO forms that show a curvature due to PLP allosteric inhibition.

## Experimental procedures

### Materials

Ingredients for bacterial growth and all reagents used for protein purification were from Sigma-Aldrich, except Phenyl-Sepharose, which was purchased from GE Healthcare. Pyridoxine 5'-phosphate was obtained from PLP (98% pure; Sigma-Aldrich) according to the method of Kazarinoff and McCormick (12).

### Site-directed mutagenesis

Mutations of the *pdxH* coding region were carried out using the pET22b (+) construct as template and a standard protocol described in the Quick-Change kit from Stratagene. For each mutation, two complementary oligonucleotide primers, synthesized by Metabion International AG, were used. The oligonucleotide sequences are listed below, and the mutated bases are underlined. *E. coli* DH5α cells were used to amplify the mutated plasmids. All mutations were confirmed by sequence analysis (Microsynth AG) of both DNA strands, and the only differences with respect to wildtype were those intended. Enzyme expression was performed using the *E. coli* MDS00 strain (12).

K145A forward, 5'-GCATGGGTTTCGGCGCAGTCCAGTCGC-3', K145A reverse, 5'-GCGACTGGACTGCGCCGAAACCCATGC-3', F177A forward, 5'-CCATTGCCGAGCGCTTGGGGCGGTTTTCGC-3', F177A reverse, 5'-GCGAAAACCGCCCCAAGCGCTCGGCAATGG-3', N84A forward, 5'-GGTGTTTTACACCGCCCTCGGCAGCCG-3', N84A reverse, 5'-CGGCTGCCGAGGGCGGTGTAAAACACC-3', N84W forward, 5'-GGTGTTTTACACCTGGCTCGGCAGCCG-3', N84W reverse, 5'-CGGCTGCCGAGCCAGGTGTAAAACACC-3', R23L forward, 5'-CAAAGGCGGGTTACTCCGCCGCGATCTTC-3', R23L reverse, 5'-GAAGATCGCGGCGGAGTAACCCGCCTTTG-3', R215L forward, 5'-GTGGAAGATTGATCTTCTTGCACCCTG-3', R215L reverse, 5'-CAGGGTGCAAGAAGATCAATCTTCCAC-3', R23L/R24L forward, 5'-GGTTACTCCTCCGCGATCTTC-3', R23L/R24L reverse, 5'-GAAGATCGCGGAGGAGTAACC-3'.

### Protein expression and purification

The *E. coli* strain MDS00 (12) was used for the protein expression. The plasmid used for the expression of the gene is a pET 22b (+) construct (Novagen). The gene was cloned in the MCS, between *NdeI* and *NcoI*. The *E. coli* strain MDS00 transformed with plasmid pET 22b(+)-PNPO was grown in LB medium supplemented with ampicillin (100 μg/ml) and chloramphenicol (34 μg/ml), at 37 °C, to an *A*_600_ of ~0.5; then, the expression of *e*PNPO was induced with 0.1 mM isopropyl-β-D-1-thiogalactopyranoside (IPTG) and the growth was continued for 12 h, at 28 °C. Cells were harvested and suspended in 50 mM potassium phosphate buffer, pH 7.3, containing 2 mM EDTA. The cell lysis was carried out by addition of lysozyme and sonication on ice, and the proteins were purified using the Bio-Gel P6DG and the CM Sephadex C-25 columns (Sigma-Aldrich), as described (13).

The apoenzyme forms were prepared at low pH through the phenyl sepharose chromatographic step, lowering the pH of the sample by adding 100 mM potassium phosphate buffer, pH 5.0, containing 5 mM 2-mercaptoethanol and 0.2 mM EDTA, as described (13).

The protein subunit concentration of both apo-PNPO and holo-PNPO was evaluated considering the contribution of FMN to the absorbance at 278 nm, using the method developed by di Salvo *et al.* (13).

### Differential scanning fluorimetry assays

DSF assays were performed using a Real Time PCR Instrument (CFX Connect Real Time PCR system, Bio-Rad). In a typical experiment, 2 μM protein in 50 mM Na-Hepes buffer, pH 7.6, containing 150 mM NaCl and 2 μM FMN, was mixed with Sypro Orange (5x, Thermo Scientific) in a total volume of 25 μl in a 96-well PCR plate. Fluorescence was measured from 25 to 95 °C in 0.4 °C/30 s steps. The excitation fluorescence was set at 450 to 490 nm, and the emission fluorescence was recorded between 560 and 580 nm. All samples were run in triplicate. Denaturation profiles were analyzed using the Graph Pad software after removal of points representing quenching of the fluorescent signal due to post-peak aggregation of protein–dye complexes. All curves were normalized and fitted to a sigmoidal equation (Equation 1) to obtain the melting temperatures (*T*_m_).

(1) $$Fluorescence=F_{1}\;\frac{\left(F_{2}\;-\;F_{1}\right)}{1\;+\;e^{T_{m}-\frac{X}{S}}}\;$$

Alternatively, the melting temperatures were obtained by plotting the first derivative of the fluorescence emission as a function of temperature (-dF/dT) by using the Bio-Rad CFX manager software.

### Determination of the dissociation constants of PLP binding equilibrium

The dissociation constants for PLP binding to wildtype and variant enzymes were analyzed taking advantage of FMN fluorescence quenching observed upon binding of this molecule to the PNPO allosteric binding site (5). Fluorescence emission measurements were carried out at 25 °C, in 50 mM Na-Hepes buffer, at pH 7.6, with a FluoroMax-3 Jobin Yvon Horiba spectrofluorimeter, using a 1-cm-pathlength quartz cuvette. All analyzed *e*PNPO forms were used at a fixed final subunit concentration of 0.1 μM, while adding increasing concentrations of PLP (from 0.1 to 6 μM). Excitation and emission slits were set at 3 and 5 nm, respectively. Dissociation constants were calculated from saturation curves obtained measuring FMN fluorescence emission intensity as a function of increasing PLP concentration and analyzed using a quadratic equation (Equation 2), in which F_rel_ is the measured relative fluorescence, F_0_ is fluorescence in the absence of ligand, F_inf_ is fluorescence at infinite ligand concentration, [P_0_] is the total PLP concentration, [E_0_] stands for the total enzyme subunit concentration, and K_D_ is the dissociation constant of the equilibrium *E + P ⇌ E·P*.

(2) $$F_{rel}=\;F_{0}+\left(F_{inf}-F_{0}\right)\times \frac{\left[P_{0}\right]+\left[E_{0}\right]\;+\;K_{D}-\sqrt{{\left(\left[P_{0}\right]+\left[E_{0}\right]+\;K_{D}\right)}^{2}-\;4\left[P_{0}\right]\left[E_{0}\right]\;}}{2\left[E_{0}\right]}$$

Titration experiments performed to determine the stoichiometry of PLP binding were performed using concentrated solutions of enzyme (2, 4, and 8 μM), using the same method, with excitation and emission slits set at 1 and 3 nm, respectively. Data were fitted using Equation 2, by fixing K_D_ at the values determined from saturation curves, so as to obtain an estimate of [E_0_].

### Kinetic studies

All kinetic measurements were performed using a Hewlett-Packard 8453 diode-array spectrophotometer (Agilent Technologies), using a 1-cm-pathlength cuvette, at 37 °C in 50 mM Tris-HCl buffer, at pH 7.6. For determination of kinetic parameters, a protein concentration of 1 μM was used with all *e*PNPO variant forms, except for the R23L/R24L PNPO variant (0.5 μM protein subunit concentration). PNP concentration was varied from 1 to 60 μM. All activity measurements were performed in the presence of a saturating FMN concentration (up to 3–6 μM) that was empirically determined for each *e*PNPO form by assaying the enzyme activity at increasing FMN concentrations. Product formation was followed at 414 nm, where the Tris-PLP aldimine product absorbs maximally with a molar absorbance coefficient of 4253 M^−1^ cm^−1^ (9). The values of *K*_M_ and *k*_CAT_ were determined from least-squares fitting of initial velocity data as a function of PNP concentration to the Michaelis–Menten equation.

Activity assay of the R23L/R24L, R23L/R215L, and R23L/R24L/R215L mutants was also carried out in 50 mM Na-Hepes buffer, at pH 7.6. In this case, PLP formation as a function of time was calculated using an extinction coefficient of 5330 M^−1^ cm^−1^ at 388 nm (5), determined using standard PLP solutions (whose concentration was determined in 0.1 M NaOH (14)). Both enzyme and PNP concentrations were fixed (0.5 and 15 μM, respectively). Reactions were started by the addition of the enzyme to buffer containing PNP and kept under constant stirring by a magnetic bar, to ensure rapid mixing. Kinetic traces were the same when the order of addition of reaction components was inverted by adding PNP last.

Inhibition kinetics were measured in 50 mM Na-Hepes pH 7.6. PLP formation was followed at 388 nm, using a molar absorbance coefficient of 5330 M^−1^ cm^−1^. All saturation curves obtained by varying PNP at a fixed PLP concentration were independently analyzed using the Michaelis–Menten equation, obtaining apparent *V*_MAX_ and apparent *K*_M_ values, which were then used to calculate 1/app*V*_MAX_ and app*K*_M_/app*V*_MAX_ as a function of PLP concentration. In order to globally analyze initial velocity and 1/app*V*_MAX_ and app*K*_M_/app*V*_MAX_ data, an equation was derived (Equation 3) on the basis of the general PLP inhibition model shown in Figure 8*A* (see Supporting information for details), which describes the initial velocity as a function of the PNP substrate (S) and the PLP product (P) concentrations:

(3) $$v=\frac{V_{MAX}}{\frac{1\;+\;\frac{\left[P\right]}{\alpha K_{I}}}{1\;+\;\beta \frac{\left[P\right]}{\alpha K_{I}}}}\;\frac{\left[S\right]}{K_{M}\frac{1\;+\;\frac{\left[P\right]}{K_{I}}\;+\;\frac{\left[P\right]}{K_{X}}\;+\;\frac{{\left[P\right]}^{2}}{K_{I}\gamma K_{X}}}{1\;+\;\frac{\left[P\right]}{\alpha K_{I}}}\;+\;\left[S\right]}$$

The same equation expresses apparent *V*_MAX_ and apparent *K*_M_ as a function of PLP (P) concentration,

$$appV_{MAX}=\frac{V_{MAX}}{\frac{1\;+\;\frac{\left[P\right]}{\alpha K_{I}}}{1\;+\;\beta \frac{\left[P\right]}{\alpha K_{I}}}}$$

$$appK_{M}=K_{M}\frac{1\;+\;\frac{\left[P\right]}{K_{I}}\;+\;\frac{\left[P\right]}{K_{X}}\;+\;\frac{{\left[P\right]}^{2}}{K_{I}\gamma K_{X}}}{1\;+\;\frac{\left[P\right]}{\alpha K_{I}}}$$

and therefore 1/app*V*_MAX_ and app*K*_M_/app*V*_MAX_ as a function of PLP (P) concentration:

(4) $$\frac{1}{appV_{MAX}}=\frac{\frac{1\;+\;\frac{\left[P\right]}{\alpha K_{I}}}{1\;+\;\beta \frac{\left[P\right]}{\alpha K_{I}}}}{V_{MAX}}$$

(5) $$\frac{appK_{M}}{appV_{MAX}}=\frac{K_{M}\frac{1\;+\;\frac{\left[P\right]}{K_{I}}\;+\;\frac{\left[P\right]}{K_{X}}\;+\;\frac{{\left[P\right]}^{2}}{K_{I}\gamma K_{X}}}{1\;+\;\frac{\left[P\right]}{\alpha K_{I}}}}{\frac{V_{MAX}}{\frac{1\;+\;\frac{\left[P\right]}{\alpha K_{I}}}{1\;+\;\beta \frac{\left[P\right]}{\alpha K_{I}}}}}$$

Initial velocity, 1/app*V*_MAX_, and app*K*_M_/app*V*_MAX_ data were globally fitted to Equations (3), (4), (5), with shared parameters.

### Measurement of PLP content of the PNPO–PLP complex

Retention of PLP by *e*PNPO was analyzed by measuring the PLP content of the protein after incubation with PLP and chromatographic separation. The PNPO variant forms (100 μM) were mixed with 100 μM PLP and incubated at 30 °C for half an hour. Unbound PLP was separated from the protein on a Superdex 200 10/300 GL SEC column (GE Healthcare Bio-Sciences), using a FPLC system. The column was equilibrated with 50 mM Na-Hepes buffer, pH 7.5. After chromatography, the PLP bound to the protein was released with 0.6 M KOH, which was neutralized with 25% v/v HClO_4_. Then, the PLP present in the PNPO samples was calculated from the activity of reconstituted holo *E. coli* serine hydroxymethyltransferase (apo-SHMT), as described (5, 15).

### Protein crystallization, data collection, and data reduction

After purification, the K72I/Y129F/R133L/H199A quadruple variant (*e*PNPOqm) was dialyzed in 100 mM potassium phosphate, pH 7.5, 5 mM 2-mercaptoethanol, 150 mM NaCl; concentrated to a final concentration of ~5 mg/ml; and finally centrifuged for 10 min at 10,000 rpm at 4 °C to remove small protein aggregates. Automated crystallization screenings were performed at 298 K with Crystal Phoenix crystallization robot (Art Robbins); consequently, the best conditions were optimized by hand. Both screening and optimization were carried out by hanging drop vapor diffusion method; the dialyzed protein solution was used to produce crystals of both *e*PNPO and *e*PNPO in complex with PLP. PLP was added to a protein solution aliquot in a 1:20 molar ratio (protein:PLP), before setting up the PLP-*e*PNPOqm plate. Optimization plates were designed with a gradient of (NH_4_)_2_SO_4_ (from 0.7 to 1.2 M), and three different ratios of protein to reservoir (1:1, 2:1, 1:2) were used to set up drops in three of four lanes, whereas in one, a fixed 1:1 ratio was used against different reservoir volumes (from 500 to 1000 μl) of a 1 M (NH_4_)_2_SO_4_ solution.

Two different crystal forms were obtained for the *e*PNPOqm and the PLP-*e*PNPOqm: the first grew up as a hexagonal bipyramid in a 2:1 drop equilibrated for 2 to 3 days against 500 μl of a 0.9 M (NH_4_)_2_SO_4_ reservoir solution, whereas the latter, grown up as a hexagonal prism, reached full size in a 1:1 drop after 5 to 7 days of equilibration against 600 μl of 1 M (NH_4_)_2_SO_4_. Crystals were fished, mounted on nylon loops, and cryoprotected by immersion in a solution containing 80% (v/v) mother liquor and 20% (v/v) glycerol, before flash-freezing in liquid nitrogen and transporting to the synchrotron radiation source. Single wavelength (λ = ~ 0.968 Å) datasets were collected at Diamond Light Source from crystals of *e*PNPOqm and PLP-*e*PNPOqm at I24 beamline, using a PILATUS3 6M detector at the temperature of 100 K. Both datasets were collected, processed, and scaled with AUTOPROC, XDS, and AIMLESS. Crystal parameters and collection statistics are available in Table 2.

### Structure determination and model refinement

Both *e*PNPOqm and PLP-*e*PNPOqm structures were solved by molecular replacement with MOLREP. The crystal structure of wildtype *e*PNPO, deprived of ligands (except FMN), waters, and ions, was used as search model (PDB code: 1G76). Structures refinement was performed with the program REFMAC5, whereas models were built in COOT.

## Data availability

All the described data are within the article, except the crystallographic data, which are deposited into the publicly accessible Protein Data Bank. The *e*PNPOqm and PLP-*e*PNPOqm structures were deposited in the Protein Data Bank with the following accession numbers: **6YLZ** (16) and **6YMH** (17), respectively.

## Supporting information

This article contains supporting information (8).

## Conflict of interest

The authors declare that they have no conflicts of interest with the contents of this article.
