# Supplementary material for: Identification and characterization of the pyridoxal 5’-phosphate allosteric site in Escherichia coli pyridoxine 5’-phosphate oxidase
Source: J Biol Chem. 2021 May 18;296:100795. doi: 10.1016/j.jbc.2021.100795 (PMC8215295; doi:10.1016/j.jbc.2021.100795)
Supplement: Supplemental Figures S1–S5 [file mmc1.pdf]

Supporting information to

Identification and characterization of the pyridoxal 5'-phosphate allosteric site in *Escherichia coli* pyridoxine 5'-phosphate oxidase

Anna Barile[1,2*], Theo Battista[1,2*], Annarita Fiorillo[1,2], Martino Luigi di Salvo[2], Francesco Malatesta[2], Angela Tramonti[1,2], Andrea Ilari[1] and Roberto Contestabile[2,#]

[1]Istituto di Biologia e Patologia Molecolari, Consiglio Nazionale delle Ricerche, P.le A. Moro, 5, 00185 Roma, Italy; [2]Istituto Pasteur Italia - Fondazione Cenci Bolognetti, Dipartimento di Scienze Biochimiche "A. Rossi Fanelli", Sapienza Università di Roma, P.le A. Moro, 5, 00185 Roma, Italy.

*These authors contributed equally to this work

[#]To whom correspondence should be addressed: Roberto Contestabile, Dipartimento di Scienze Biochimiche "A. Rossi Fanelli", Sapienza Università di Roma, Piazzale Aldo Moro 5, 00185 Roma, Italy; roberto.contestabile@uniroma1.it; Tel (+39) 0649913176.

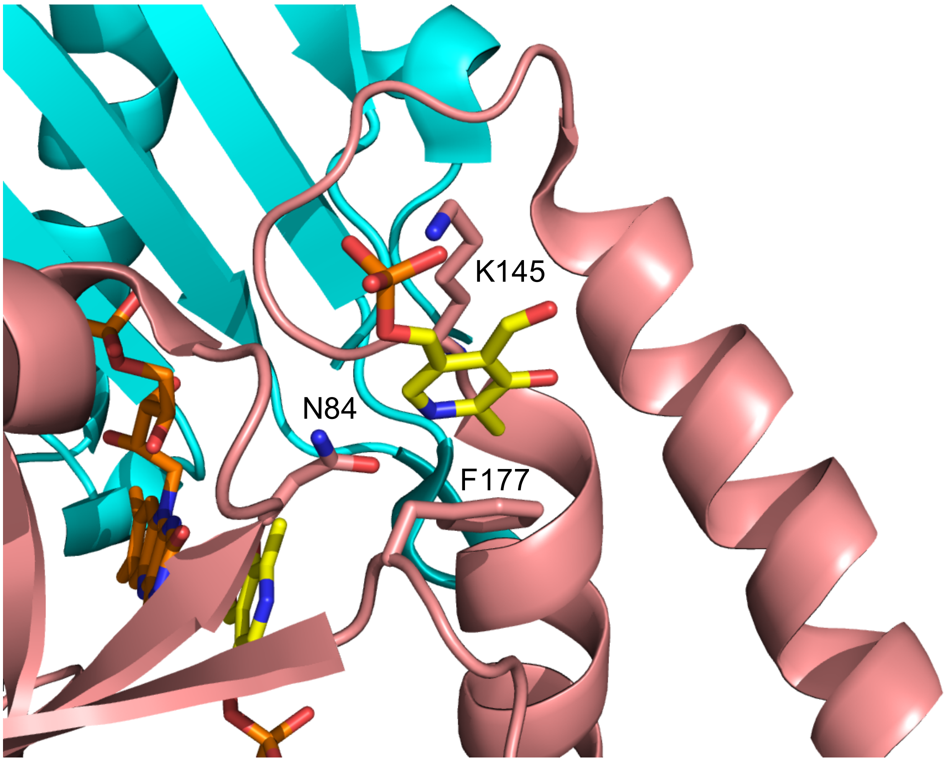

Figure S1. Putative secondary PLP binding site of *e*PNPO*.* Crystal structure of *e*PNPO obtained by Safo et al. 2001 (*J. Mol. Biol.* 310, 817-826; PDB code: 1G79) by soaking native crystals of the protein in a solution containing 40 mM PLP. The figure shows the PLP molecule (yellow sticks) in the higher occupancy position (see text for details) bound at the protein surface, about 11 Å from the active site, interacting with residues N84, K145 and F177. FMN (orange sticks) and PLP bound at the active site are visible in the background.

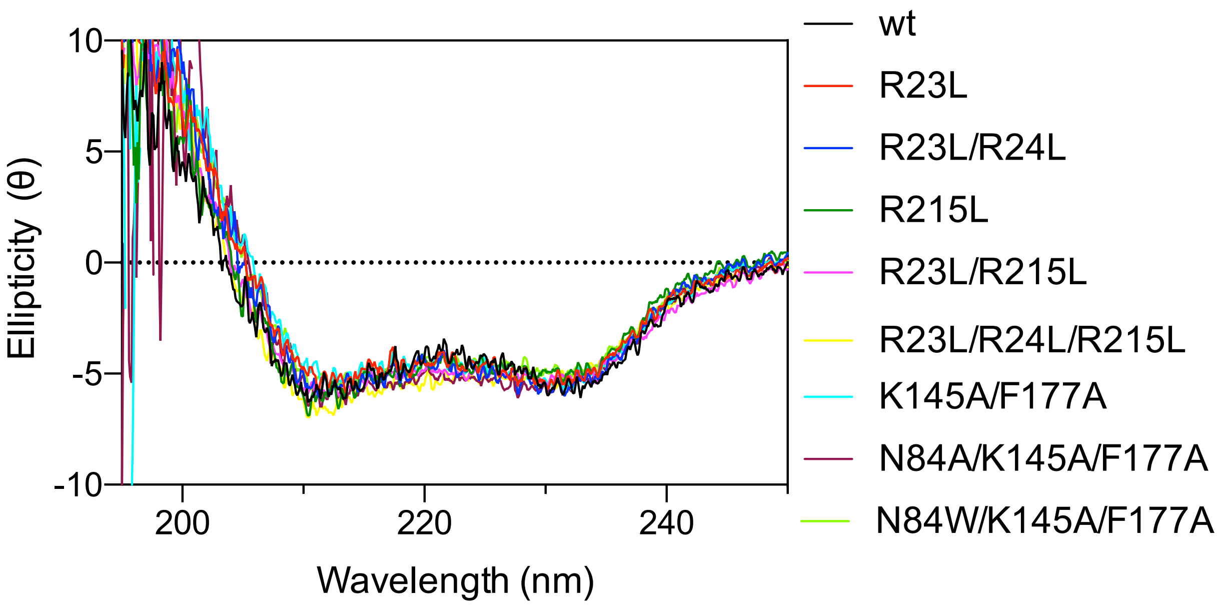

Figure S2. CD spectra of *e*PNPO mutants*.* Far UV CD spectra were measured in 50 mM Na-HEPES buffer, pH 7.6.

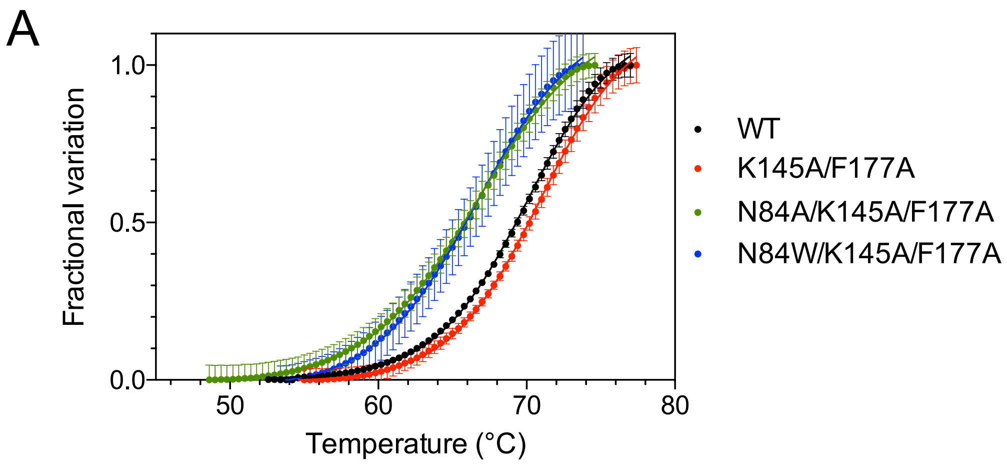

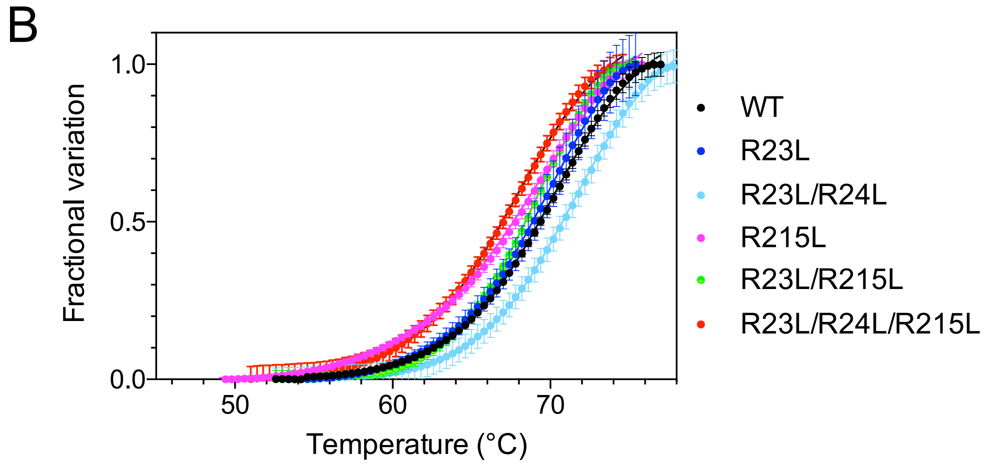

Figure S3. Differential scanning fluorimetry measurements carried out with *e*PNPO mutants. Fluorescence changes, expressed as fractional variation as a function of temperature, obtained with wild type and mutant *e*PNPO forms of the putative secondary sites indicated by previous crystallographic studies (A), and of the allosteric PLP binding site identified in this study (B). Enzyme solutions (2 μM) were analysed in 50 mM Na-HEPES buffer, pH 7.6, containing 150 mM NaCl and 2 μM FMN. All data were fitted to a sigmoidal equation (Eq. 1), obtaining the continuous lines through the experimental points (average ± standard error of three independent measurements) and the melting temperatures (T_m) values reported in (Table 1).

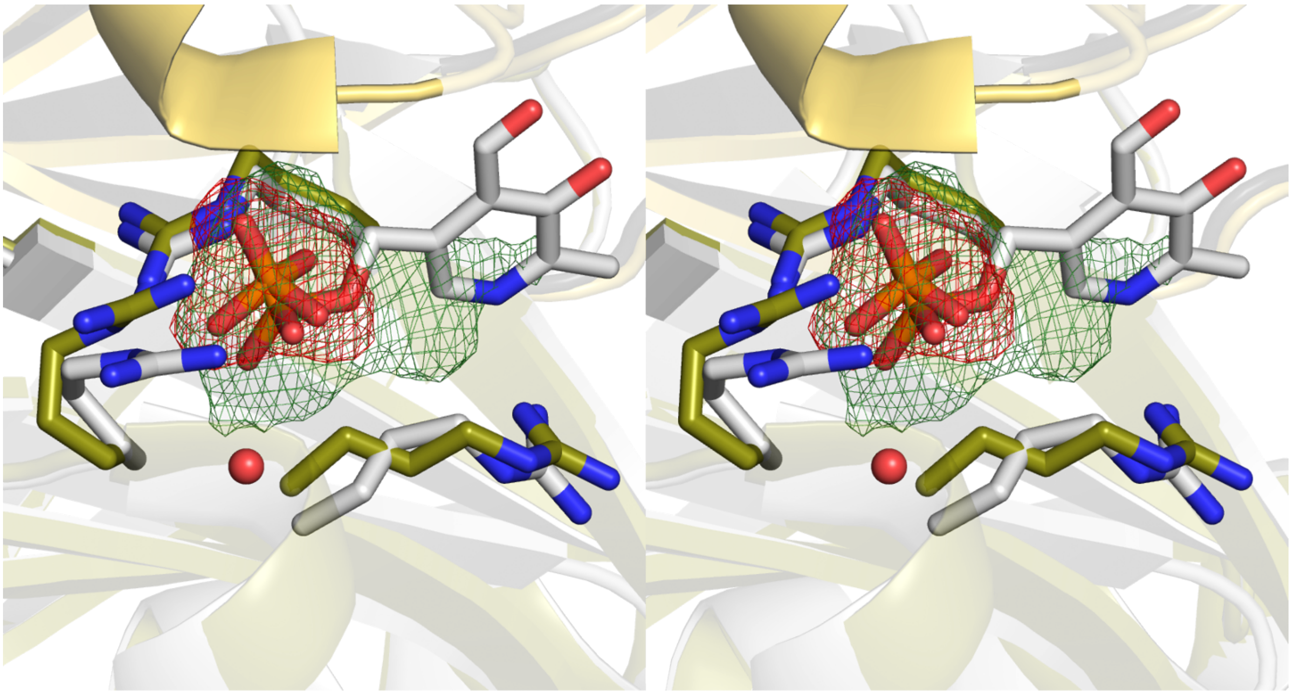

Figure S4. Stereoview of the Arg-cage of the superimposed wild type *e*PNPO (gold) (PDB code: 1G79) and PLP bound *e*PNPOqm (grey). In the figure, the Fo – Fc omit maps contoured at 2.5 σ (green for PLP, contained in the PLP-*e*PNPOqm, red for phosphate, contained in the 1G79 structure) are represented. The side chains of arginine residues lining the cage, the phosphate ion and PLP are represented as sticks.

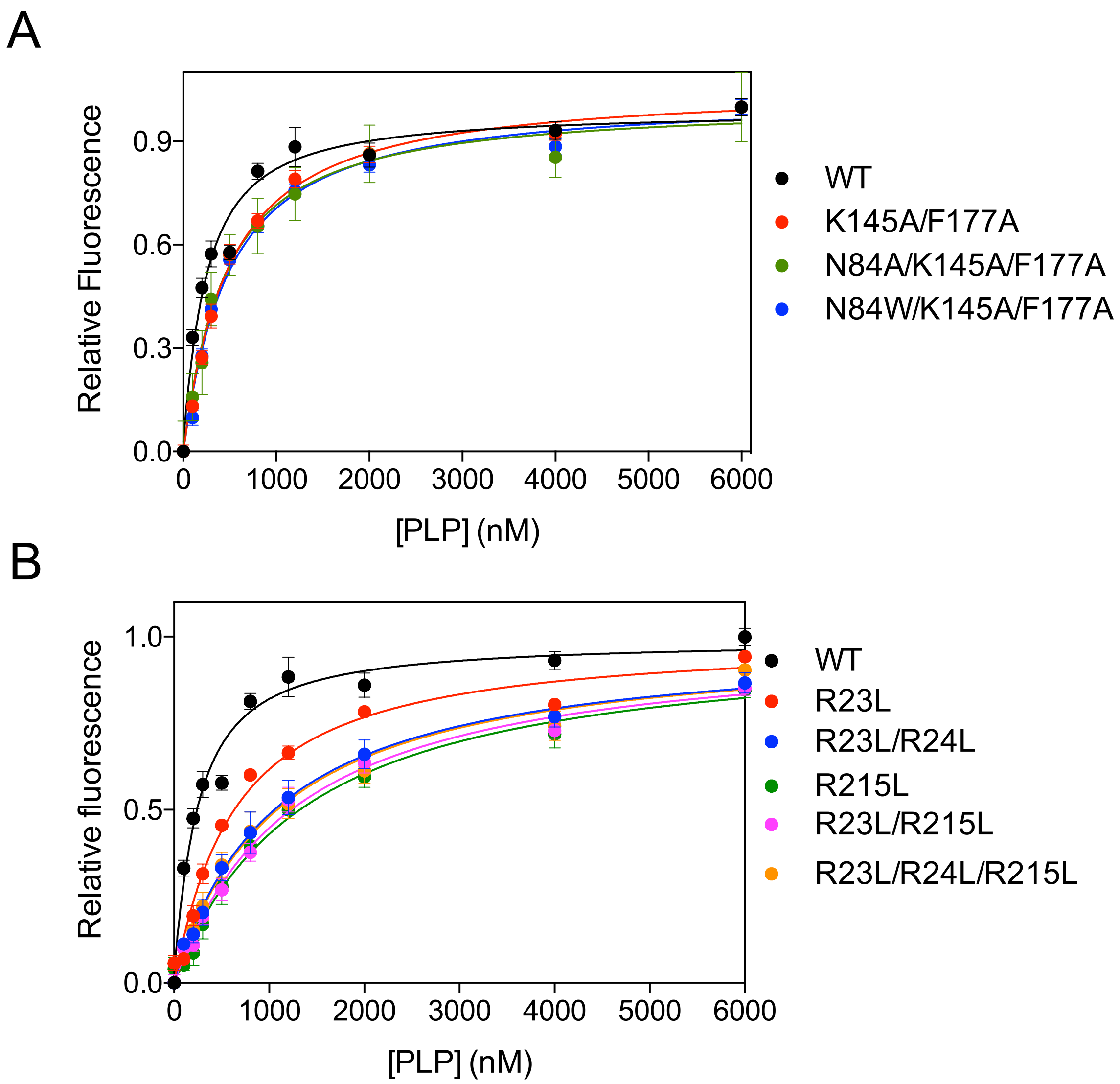

Figure S5. Analysis of PLP binding equilibrium of *e*PNPO mutants. PLP binding curve obtained with 100 nM PNPO (protein subunit concentration) wild type and mutants indicated by previous crystallographic studies (A) and mutants of Arginines (B). Emission spectra (from 470 and 570 nm) of the different *e*PNPO forms in the presence of different PLP concentrations were measured in 50 mM NaHEPES, pH 7.6, upon excitation at 450 nm. The average relative fluorescence emission between 520 and 530 nm as a function of total PLP concentration was analyzed with Eq. 2 (Experimental procedures) giving the dissociation constants reported in Table 1. Reported data are the average ± standard deviation of three independent measurements.

Derivation of the equations (Eq. 3, Eq. 4 and Eq. 5) used to globally fit initial velocity, 1/appV_{MAX} and appK_M/appV_{MAX} data shown in Fig. 5.

From Scheme 2A,

$$v = k_{CAT}[ES] + \beta k_{CAT}[PES]$$

$$\frac{v}{[E_T]} = \frac{k_{CAT}[ES] + \beta k_{CAT}[PES]}{[E] + [ES] + [EP] + [PE] + [PEP] + [PES]}$$

Given that

$$K_X = \frac{[E][P]}{[EP]}\ ; \gamma K_X = \frac{[PE][P]}{[PEP]}; K_I = \frac{[E][P]}{[PE]}; \alpha K_I = \frac{[ES][P]}{[PES]};\ \gamma K_I = \frac{[EP][P]}{[PEP]}; K_M = \frac{[E][S]}{[ES]}; \alpha K_M = \frac{[PE][S]}{[PES]}$$

then

$$\frac{v}{[E_T]} = \frac{k_{CAT}\frac{[E][S]}{K_M} + \beta k_{CAT}\frac{[E][S][P]}{K_M \alpha K_I}}{[E] + \frac{[E][S]}{K_M} + \frac{[E][P]}{K_I} + \frac{[E][P][S]}{K_I \alpha K_M} + \frac{[E][P]}{K_X} + \frac{[E][P]^2}{K_I \gamma K_X}}$$

Since [E] can be eliminated and $[E_T] = \frac{V_{MAX}}{k_{CAT}}$, then

$$\frac{v}{V_{MAX}} = \frac{\frac{[S]}{K_M} + \beta\frac{[S][P]}{K_M \alpha K_I}}{1 + \frac{[S]}{K_M} + \frac{[P]}{K_I} + \frac{[P][S]}{K_I \alpha K_M} + \frac{[P]}{K_X} + \frac{[P]^2}{K_I \gamma K_X}}$$

Multiplying by $\frac{K_M}{K_M}$

$$\frac{v}{V_{MAX}} = \frac{[S] + \beta\frac{[S][P]}{\alpha K_I}}{K_M + [S] + \frac{K_M[P]}{K_I} + \frac{[P][S]}{\alpha K_I} + \frac{K_M[P]}{K_X} + \frac{K_M[P]^2}{K_I \gamma K_X}}$$

grouping for [S] and K_M,

$$\frac{v}{V_{MAX}} = \frac{[S]\left(1+\beta\frac{[S][P]}{\alpha K_I}\right)}{K_M\left(1+\frac{[P]}{K_I}+\frac{[P]}{K_X}+\frac{[P]^2}{K_I\gamma K_X}\right)+[S]\left(1+\frac{[P]}{\alpha K_I}\right)}$$

and dividing by $\left(1+\beta\frac{[P]}{\alpha K_I}\right)$

$$v = V_{MAX}\frac{[S]}{K_M\left(\frac{1+\frac{[P]}{K_I}+\frac{[P]}{K_X}+\frac{[P]^2}{K_I\gamma K_X}}{\left(1+\beta\frac{[S][P]}{\alpha K_I}\right)}\right)+[S]\left(\frac{1+\frac{[P]}{\alpha K_I}}{\left(1+\beta\frac{[S][P]}{\alpha K_I}\right)}\right)}$$

This can be converted in the Michaelis-Menten form of Eq. 3.

$$v = \frac{V_{MAX}}{\frac{1+\frac{[P]}{\alpha K_I}}{1+\beta\frac{[P]}{\alpha K_I}}}\ \frac{[S]}{K_M\frac{1+\frac{[P]}{K_I}+\frac{[P]}{K_X}+\frac{[P]^2}{K_I\gamma K_X}}{1+\frac{[P]}{\alpha K_I}}+[S]}$$

The same equation expresses apparent V_{MAX} and apparent K_M as a function of PLP (P) concentration,

$$appV_{MAX} = \frac{V_{MAX}}{\frac{1+\frac{[P]}{\alpha K_I}}{1+\beta\frac{[P]}{\alpha K_I}}} \qquad appK_M = K_M\frac{1+\frac{[P]}{K_I}+\frac{[P]}{K_X}+\frac{[P]^2}{K_I\gamma K_X}}{1+\frac{[P]}{\alpha K_I}}$$

and therefore $1/appV_{MAX}$ and $appK_M/appV_{MAX}$ as a function of PLP (P) concentration:

$$\frac{1}{appV_{MAX}} = \frac{\frac{1+\frac{[P]}{\alpha K_I}}{1+\beta\frac{[P]}{\alpha K_I}}}{V_{MAX}} \quad \text{(Eq. 4)}$$

$$\frac{appK_M}{appV_{MAX}} = \frac{K_M\frac{1+\frac{[P]}{K_I}+\frac{[P]}{K_X}+\frac{[P]^2}{K_I\gamma K_X}}{1+\frac{[P]}{\alpha K_I}}}{\frac{V_{MAX}}{\frac{1+\frac{[P]}{\alpha K_I}}{1+\beta\frac{[P]}{\alpha K_I}}}} \quad \text{(Eq. 5)}$$
